# Metal-Based Categorization of Recent Metal Organic Frameworks with Application in Bone Disease Treatment

**DOI:** 10.1021/acsbiomaterials.5c00830

**Published:** 2025-10-31

**Authors:** Mojdeh Savadkouhi Ghoudjanaki, Fatemeh Bahadorani, Mahnaz Hosseini, Ritu Panta, Benson Karimi

**Affiliations:** † Department of Chemistry, 48456 Isfahan University of Technology, Isfahan 84156–83111, Iran; ‡ Department of Physical and Environmental Sciences, 14736 Texas A&M University Corpus Christi, Corpus Christi, Texas 78412, United States

**Keywords:** metal organic Framework, bone defect, metal ions, bone regeneration, orthopedic diseases, osteogenesis

## Abstract

Recent advancements in metal–organic frameworks (MOFs) have transformed the field of bone disease treatment by offering multifunctional platforms that integrate targeted drug delivery, enhanced osteogenesis, and antibacterial properties within a single material system. This review categorizes contemporary MOFs based on their metal constituents, such as zinc, magnesium, zirconium, cobalt, calcium, iron, copper, titanium, and rare earth metals, emphasizing their distinct physicochemical properties and unique biological functionalities. Unlike traditional biomaterials, MOFs provide highly tunable pore architectures and exceptional surface areas for efficient drug encapsulation and controlled release, facilitating localized therapeutic effects with minimal invasiveness. Cutting-edge developments include one-pot synthesis methods that enable the uniform distribution of therapeutic agents and sustained release profiles, significantly improving clinical applicability. This perspective highlights the synergistic effects of MOFs combined with scaffolds, hydrogels, and implants, which promote cellular proliferation, osteogenic differentiation, and angiogenesis, thus addressing critical challenges in bone regeneration. Moreover, emerging insights into metal ion-specific mechanisms such as calcium signaling in osteogenesis, zinc-mediated angiogenesis, and the antibacterial role of copper and rare earth elements underscore the strategic design of MOFs tailored to complex bone pathologies, including osteoporosis, infections, and osteosarcoma. This comprehensive overview not only maps recent progress but also delineates future research directions to optimize MOF functionality and expedite its translation into clinical bone therapies.

## Graphical abstract

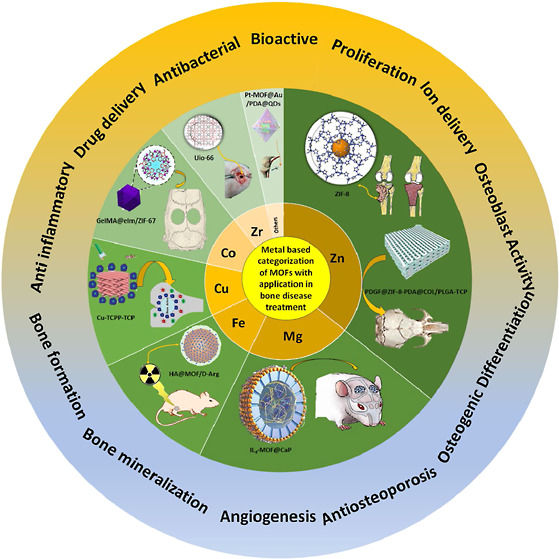

## 1 Introduction

Bone defects, which may arise from trauma, infection, neoplasms, functional atrophy, or congenital abnormalities, substantially impair patients’ quality of life by causing pain, reduced mobility, and compromised function.^1^ Effectively managing these defects remains a critical challenge in orthopedics and regenerative medicine. Conventional therapeutic strategies include autografts, allografts, bone substitute materials, and tissue engineering approaches.^2^

Autologous bone grafts are considered the clinical gold standard due to their intrinsic osteogenic capacity and excellent.^3,4^ However, limitations such as the restricted availability of graft material, donor-site morbidity, and possible complications restrict their widespread application. Allogeneic grafts—harvested from cadaveric donors—offer greater availability but lack adequate osteoinductive properties and carry potential risks of immunogenicity and pathogen transmission.^5–7^

Bone substitute materials are broadly categorized into ceramics, metals, polymers, composites, and natural biomaterials, each characterized by distinct physicochemical and biological properties.^8,9^ Calcium phosphate ceramics emulate the mineral phase of native bone and demonstrate favorable biocompatibility.^10,11^ Metallic materials, including titanium and its alloys, provide superior mechanical strength and stability.^12^ Polyglycolic acid and polylactic acid, two biodegradable polymers, offer versatility and scaffolding functions conducive to tissue regeneration.^13^ Composite materials combine attributes of multiple constituents to enhance mechanical and biological performance.^14^ Natural biomaterials, such as collagen and hydroxyapatite-based scaffolds, inherently support cellular adhesion and proliferation due to their bioactivity.^15^

Despite these advantages, each material class possesses inherent limitations. For instance, ceramics may exhibit brittleness and insufficient fracture toughness;^16^ metals may induce stress shielding and corrosion;^17^ polymers often degrade prematurely or provoke inflammatory responses;^18^ composites demand complex fabrication processes;^19^ and natural biomaterials may suffer from scarcity. Addressing these challenges is imperative for the optimization of bone substitute performance.

Recent innovations in drug delivery systems and implants have facilitated localized, controlled administration of therapeutics, thereby enhancing bone regeneration while minimizing invasive procedures.^20^ Within this landscape, MOFs have surfaced as highly promising candidates owing to their distinctive structural and functional characteristics suitable for targeted drug delivery in bone disease treatment.^21^

MOFs such as ZIF-8, UiO-66, and MIL-101 illustrate remarkable porosity, thermal stability, and selective adsorption capabilities, enabling versatile biomedical applications.^22–24^ Their extensive surface area and adjustable pore environments support efficient drug loading and sustained release, rendering MOFs valuable as bone substitute materials.^22,25^

In the field of medicine, they have garnered significant attention owing to their potential as bone substitutes for treating bone diseases. Their unique properties, including a large surface area for drug loading, biocompatibility, and controlled release capabilities, make them promising candidates for the repair and regeneration of bone defects.^26^

Moreover, MOFs can recapitulate components of the bone mineral composition and undergo surface modifications to augment their suitability for bone regeneration. Their incorporation into biomaterial scaffolds imparts mechanical support while facilitating osteoconduction and cellular guidance, culminating in a multifaceted approach to bone defect management.^27,28^ Besides, MOFs can serve as carriers for therapeutic agents, enabling localized and sustained drug delivery to promote bone healing.^29^ Additionally, the ability of MOFs to emulate the mineral composition of bone and their potential for surface modification further enhance their suitability as bone substitute materials.^26^

The incorporation of metal ions within MOFs confers additional therapeutic functionalities, including osteogenic stimulation, osteoclastic inhibition, angiogenesis promotion, and antimicrobial activity. Consequently, ion-modified MOFs represent multifunctional platforms for bone disease treatment.^30^

This review delineates the evolving role of MOFs in the field of bone tissue engineering and emphasizes their potential to overcome the complex clinical challenges posed by bone defects.

## 2 Fundamental Concepts

### 2.1 Bone Structure

A thorough comprehension of the hierarchical structure of bone and its regenerative mechanisms is essential for the logical design of MOF-based therapeutic strategies. The bone matrix comprises approximately 30% organic components and 70% inorganic minerals by weight. Collagen fibers constitute nearly 90% of the organic fraction, with the remainder comprising noncollagenous proteins, proteoglycans, lipids, osteopontin, and other matrix molecules that contribute to mechanical resilience and tissue adhesion (Figure 1). The mineral phase predominantly consists of hexagonal hydroxyapatite crystals. Electrostatic interactions involving calcium (Ca^2+^) and phosphate (PO_4_^3–^) ions enable crystal binding to the organic matrix. These hydroxyapatite crystals are organized in parallel alignment with the longitudinal axis of collagen fibers via the self-assembly of collagen triple helices, thereby conferring structural integrity and mechanical strength.^31,32^

**Figure 1:**
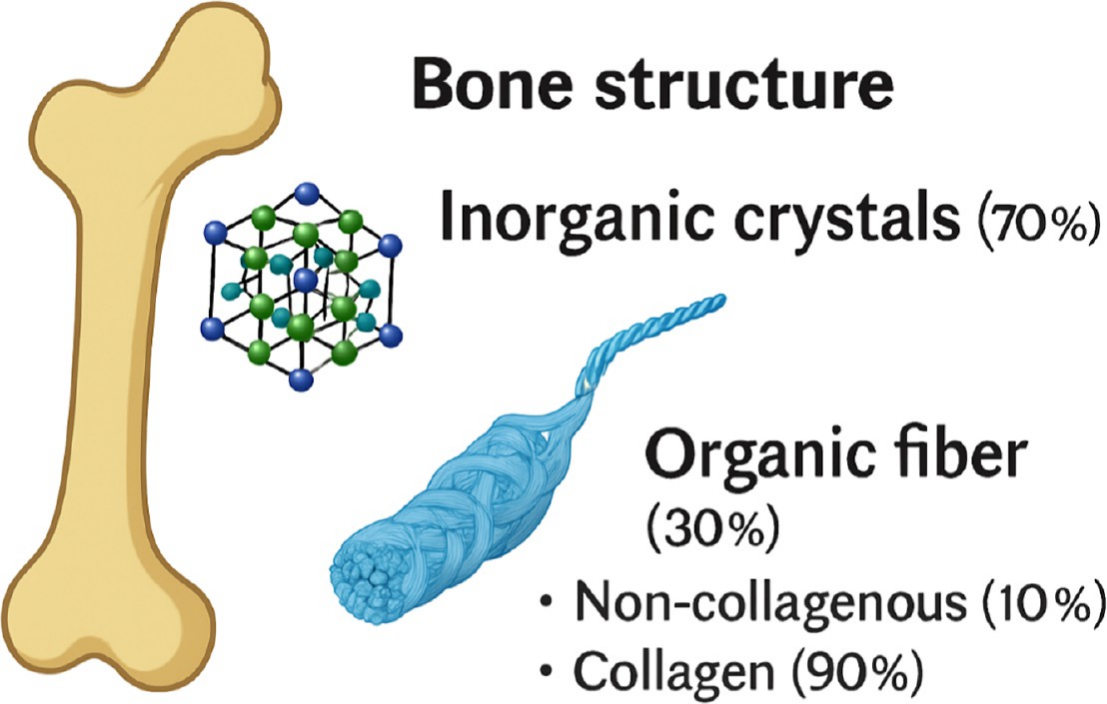
Schematic illustration of the bone structure.

### 2.2 Mechanism of Bone Regeneration

In contrast to other tissues, it has been discovered that bone repair recapitulates ontogenetic developmental processes, enabling restoration of preinjury composition, architecture, and function.^33^ The healing cascade is initiated by an inflammatory phase, lasting several days, which leads to hematoma formation and clot stabilization at the defect. This phase is succeeded by the recruitment and differentiation of mesenchymal stem cells into osteoblasts and endothelial cells, promoted by osteogenic growth factors. Mineral deposition ensues over a 21- to 35-day period, whereby soft callus is replaced by woven bone, followed by remodeling into lamellar bone, restoring native functionality.^31^

### 2.3 Bone Diseases and Therapeutic Strategies

Although bone exhibits intrinsic regenerative capacity, large-scale injuries resulting from osteosarcoma, congenital deformities, infectious etiologies, aging, or trauma often exceed physiological repair capability, necessitating the use of artificial bone substitutes. Optimal biomaterials must satisfy criteria including biocompatibility, biodegradability, hydrophilicity, nontoxicity, and antibacterial efficacy.^34^

Distinct pathologies impose specific therapeutic requirements; for instance, sarcomatous lesions demand simultaneous tumor suppression and facilitation of bone regeneration, while chronic inflammatory conditions such as rheumatoid arthritis necessitate materials possessing anti-inflammatory and joint repair capabilities.^35^

Various biomaterials, including bioactive glasses, growth factor-laden microspheres, polymeric scaffolds with hydroxyapatite reinforcement, surface-modified implants, and calcium phosphate nanoparticles, have been investigated.^34^ Despite incremental progress, an ideal “gold standard” material remains elusive, and MOFs have attracted significant interest owing to their distinctive physicochemical characteristics and multifunctionality.

## 3 MOF Synthesis and Design for Bone Regeneration

### 3.1 Metal–Organic Frameworks

Metal–organic frameworks (MOFs) have appeared as a fascinating category of porous materials with varied structures and tunable properties, garnering significant attention in various fields of research. MOFs formed by the coordination of metal ions or clusters with polytopic ligands (inorganic or organic linkers), resulting in high surface areas, large pore volumes, and exceptional chemical versatility.^36,37^ MOFs can be broadly classified into several unique types according to their metal clusters, metal ions, organic ligands, and synthesis methods. MOFs are typically sorted into distinct subfamilies based on their metal centers and organic linkers, including isoreticular MOFs (IR-MOFs), Materials of Institute Lavoisier (MIL) frameworks, zeolitic imidazolate frameworks (ZIFs), and University of Oslo (UiO) frameworks.^36,38^

The utilization of MOFs as bone substitutes offers distinct advantages over other treatment methods, highlighting their superiority in addressing bone diseases. MOFs possess unique physical and mechanical properties that enhance their effectiveness in the processes of bone repair and regeneration. Compared to traditional bone grafting, MOFs provide a more versatile platform for drug delivery and targeted release, enabling localized treatment and reducing systemic side effects.^39^ Additionally, MOFs exhibit outstanding anticorrosion properties, ensuring durability and long-term stability of the implant. Furthermore, the porous network and postmodification potential of MOFs make them ideal substances for targeting damaged areas and promoting the proliferation and adhesion of bone cells, thus facilitating beneficial bone regeneration.^40^ In terms of anti-infection capabilities, MOFs can be functionalized with antimicrobial agents, preventing bacterial colonization and decreasing the risk of implant-associated infections.^41^ Biodegradable polymers, although widely used, may degrade too quickly, while MOFs offer controlled degradation rates, allowing for proper tissue regeneration without compromising mechanical integrity.^42^ Overall, MOFs outperform other methods in terms of drug release and delivery, targeted delivery, anticorrosion properties, anti-infection capabilities, and biocompatibility, making them a promising alternative for treating bone diseases.

### 3.2 Synthesis Methods

The synthesis of MOFs encompasses various techniques, each presenting distinct benefits regarding controllability, scalability, and structural diversity (Figure 2).^43^ All of these methods allow for the easy synthesis of porous MOF materials that possess highly complex yet well-ordered structural networks. They preserve the distinctive composition of their constituent components and ensure the high reproducibility of forming materials with the desired crystallinity. The primary synthesis methods for MOFs include conventional synthesis methods, the solvothermal and hydrothermal methods,^44^ microwave-assisted synthesis, electrochemical synthesis, mechanochemical synthesis, sonochemical synthesis, and vapor-phase deposition.^45–47^ The selection of a specific synthesis method has significant implications for the design of MOFs, as various approaches directly affect the structural properties, functionality, and scalability of the resulting materials. In the subsequent sections of this section, we discuss various aspects of the documented synthesis techniques for disparate porous MOF systems, emphasizing the benefits and challenges associated with each approach.

**Figure 2:**
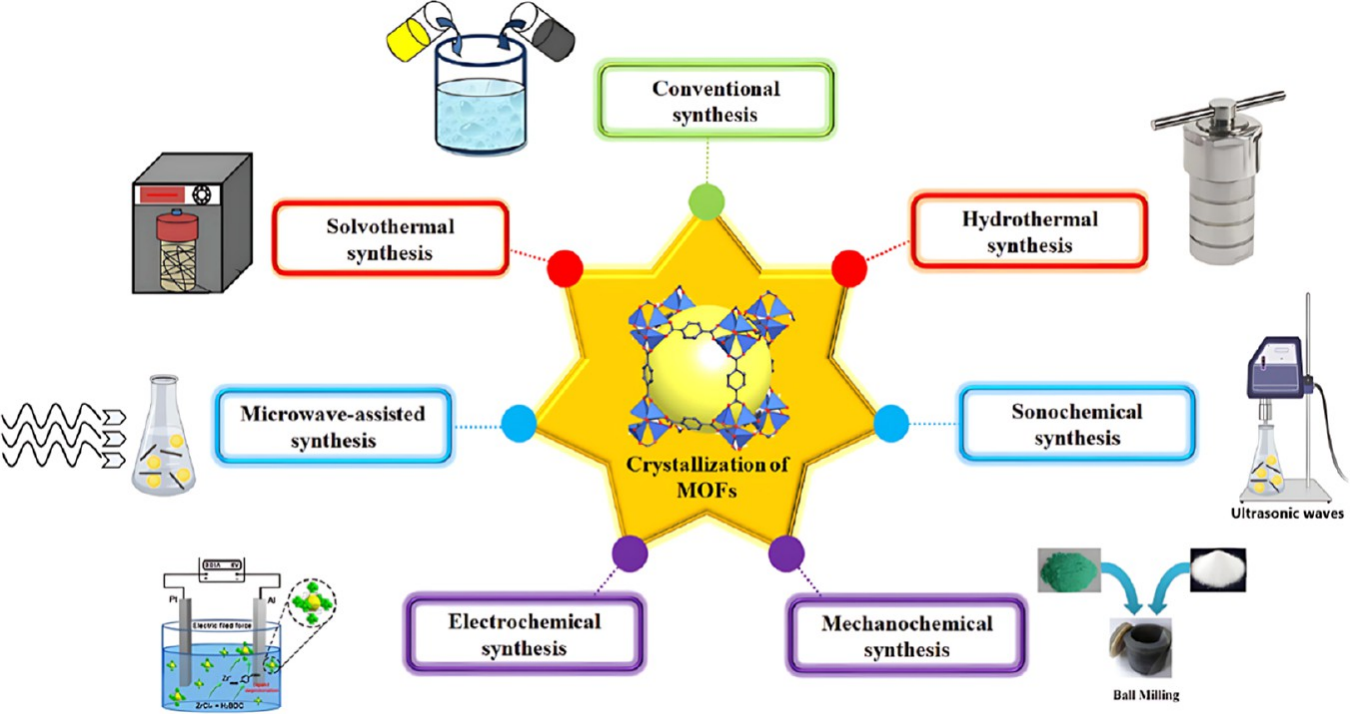
Different methods for the synthesis of MOF.

#### 3.2.1 Conventional Synthesis Method (Precipitation Technique)

One of the most ordinary methods for synthesizing MOFs is the conventional synthesis method. This technique offers several advantages, including higher yields, reduced energy requirements, and a significantly faster synthesis process. It utilizes a poor solvent that triggers the precipitation of the MOF. There are generally two simple methods to accomplish this. In one method, organic linkers and metal ions are dissolved in separate solvents before being combined, resulting in the formation and precipitation of the MOF. In the second method, both precursors are initially dissolved in a single solvent, which is subsequently transferred to another solvent that promotes precipitation. By adjustment of factors such as the concentration of precursors, the pH of the solution, and the selection of the precipitating solvent, the crystallization of the resulting MOFs can be controlled. The primary aim of these adjustments is to manage the solubility of both the starting materials and the final MOF particles.

#### 3.2.2 Hydrothermal and Solvothermal Synthesis Methods

Hydrothermal and solvothermal methods are among the most commonly used techniques for producing MOFs. These approaches involve dissolving organic linkers and metal salts in a suitable solvent, either organic (solvothermal) or water (hydrothermal), and heating the mixture within a sealed container at temperatures generally exceeding the boiling point of the solvent. This is usually done under autogenous pressure inside specialized reaction vessels such as PTFE-lined autoclaves, pressure-resistant bottles, glass flasks, glass vials, or Schlenk bottles. The combination of elevated temperature, pressure, and solvent conditions facilitates the dissolution of the reactants, allowing for the slow and controlled formation of high-quality MOF crystals with precise pore structures and high crystallinity.

One of the main benefits of these methods is that the reaction conditions, especially the temperature, pressure, time, and cooling rate, can be carefully adjusted to tailor the particle size, shape, and overall framework topology. The slow, reversible coordination of metal ions with multitopic organic linkers helps to correct any bonding errors during synthesis, resulting in well-ordered crystalline networks. However, solvothermal and hydrothermal syntheses often require long reaction times, ranging from hours to days and, in the case of solvothermal methods, frequently rely on organic solvents.

Different reaction vessels serve specific needs. For example, glass vials are inexpensive and allow visual monitoring of crystal growth but may not withstand high temperatures and pressures, especially when sealed with plastic lids. Vials with metal lids improve the temperature resistance but are costlier. Glass flasks enable larger-scale synthesis but are typically not sealed, limiting them to reactions under atmospheric pressure. Pressure-resistant bottles can endure higher pressures but require careful estimation of the internal pressure to ensure safe operation. Schlenk bottles are useful when working with air- or moisture-sensitive compounds since they maintain low-humidity, oxygen-free conditions but generally operate at ambient pressure. PTFE-lined autoclaves are among the most common vessels for solvothermal and hydrothermal synthesis, because they can safely handle high temperatures and pressures and are resistant to corrosive solvents and chemicals.

Overall, while solvothermal and hydrothermal approaches reliably produce MOFs with high structural integrity and well-defined features, the choice of vessel, solvent, and parameters of the reaction must be meticulously evaluated to attain the intended material characteristics and synthesis efficiency. In addition, it is unfavorable for scaling up due to harsh synthesis conditions.

#### 3.2.3 Microwave-Assisted Synthesis

Microwave-assisted synthesis has emerged as a potent and effective technique for the preparation of MOFs. This technique relies on dielectric heating, where microwave radiation interacts with polar molecules, primarily the solvent, causing them to oscillate rapidly. This molecular movement produces heat consistently and rapidly across the reaction mixture, greatly expediting the synthesis process in comparison with traditional heating techniques.

In a typical setup, the metal precursors and organic ligands are dissolved in a suitable solvent and sealed in a Teflon-lined vessel. When exposed to microwave energy, the solvent absorbs the radiation, resulting in a rapid temperature increase, often surpassing the boiling point of the solvent. This promotes fast nucleation and growth of crystals, enabling the formation of MOFs in a matter of minutes rather than days.

Microwave heating offers several advantages such as fast reaction times, uniform heating, energy efficiency, and controlled morphology. Despite often producing smaller crystals than traditional methods, microwave-assisted synthesis typically yields materials with a high surface area and purity. It is also well-suited for high-throughput screening of various metal–ligand–solvent systems, accelerating the identification of novel MOFs with desired properties.

Overall, microwave-assisted synthesis stands out as a fast, scalable, and versatile alternative to conventional techniques, especially for applications requiring precise control over material properties and rapid production.^44^

#### 3.2.4 Sonochemical Synthesis Method

Sonochemical synthesis, also known as ultrasonic synthesis, is an efficient alternative to conventional solvothermal methods for fabricating MOFs. This technique uses high-frequency ultrasonic waves to induce acoustic cavitation—a process where microscopic bubbles repeatedly form, grow, and violently collapse in a liquid medium. The collapse of these bubbles generates localized high temperatures and pressures, creating transient “hot spots” that drive rapid chemical reactions. These intense conditions promote the formation of reactive species such as free radicals, which accelerate the synthesis of high-quality MOFs with smaller crystal sizes and higher yields. Compared with traditional methods, ultrasound-assisted synthesis greatly shortens reaction times, often completing in minutes rather than hours, and offers improved energy efficiency and simplicity. Studies have shown that reaction duration significantly affects the morphology of the resulting crystals: shorter sonication can yield tiny spherical nanoparticles, while longer exposure produces larger, needle-like structures. Overall, the cavitation effect not only speeds up the reaction kinetics but also improves the crystallinity and uniformity of MOFs, making ultrasonic synthesis an environmentally friendly and effective approach for producing advanced porous materials.

#### 3.2.5 Electrochemical Synthesis

Electrochemical synthesis has emerged as a practical and versatile method for producing a broad spectrum of MOFs. This approach relies on the application of an electric current to an electrolyte solution, which drives the controlled assembly of organic ligands and metal ions. Electrochemical synthesis can be carried out through anodic dissolution, where metal ions are released directly from the electrode by oxidation, or through cathodic deposition, which locally raises the pH and promotes ligand coordination. This method eliminates the need for metal salts, avoiding corrosive anions that can interfere with crystal growth, and typically operates at temperatures lower than those of traditional solvothermal methods.

Notably, electrochemical techniques offer precise control over ion concentration, reaction rates, and crystal morphology, resulting in MOFs with high purity, excellent crystallinity, and smaller crystal sizes. The method’s mild conditions, ease of operation, and energy efficiency make it an attractive alternative, especially for producing coatings and thin films on conductive substrates useful for applications like energy storage, sensors, and catalysis. Although organic solvents are often used, which can influence surface area and stability, the electrochemical approach remains an innovative, sustainable, and controllable route for engineering MOFs with tailored structures and properties.

#### 3.2.6 Mechanochemical Synthesis

Mechanochemical synthesis, which relies on the application of mechanical force to facilitate chemical reactions, has become an encouraging and sustainable alternative for producing MOFs. This method typically uses milling or grinding, often with a ball mill or simple mortar and pestle, to activate solid reactants through forces such as compression, shear, and friction. The repeated impact breaks crystallographic bonds, generates fresh reactive surfaces, and induces structural changes that promote the formation of new MOF structures. Mechanochemical approaches stand out for being solvent-free or requiring minimal solvents, conforming effectively to the principles of green chemistry by minimizing hazardous waste and energy requirements.

Overall, mechanochemical synthesis offers significant advantages over conventional methods, such as shorter reaction times (often just 10–60 min), lower temperatures, high yields, and the ability to produce MOFs with novel properties. As research advances, mechanical grinding continues to drive sustainable and cost-effective pathways for developing advanced porous materials on an industrial scale.

In summary, the choice of the synthesis method is influenced by several factors, including the desired MOF properties, scalability requirements, and the specific application in bone disease treatment.^48–51^

## 4 Discussion

### 4.1 Hero Role of Metal Ions in Bone Regeneration

In the design and synthesis of MOFs for the treatment of bone diseases, the meticulous selection of metal ions is paramount due to their distinct properties and biological functions. A variety of metal ions have been employed to engineer MOF structures with tailored characteristics to address specific therapeutic objectives. Calcium ions (Ca^2+^) are frequently incorporated into MOFs to replicate the natural bone composition, thereby enhancing biocompatibility and promoting biomineralization.^52^ Zinc ions (Zn^2+^), recognized for their integral role in bone metabolism and wound healing, are utilized within MOFs to support osteogenesis and modulate bone cell activities.^53^ Copper ions (Cu^2+^), owing to their antibacterial and angiogenic effects, have been integrated into MOFs to combat bone infections and improve vascularization during tissue regeneration.^54^ Magnesium ions (Mg^2+^), essential for bone formation and structural integrity, contribute to MOFs by enhancing mechanical strength, stimulating osteoblast function, and regulating bone remodeling processes.^55^ Additionally, strontium ions (Sr^2+^) have demonstrated efficacy in MOFs by promoting bone formation and inhibiting resorption, rendering them promising for osteoporosis treatment. Other metal ions, including silver (Ag^+^), gold (Au^3+^), iron (Fe^3+^), cobalt (Co^3+^), manganese (Mn^2+^), and titanium (Ti^4+^), have been investigated for their antimicrobial, antioxidant, and osteogenic properties, thereby broadening the therapeutic potential of MOFs in managing bone diseases.^53–56^ The subsequent tables provide a comparative analysis of the roles of these metals in bone regeneration.

#### 4.1.1 The Role of Zn^2+^ and Zn-Based MOFs in Bone Regeneration

Zinc ions (Zn^2+^) and zinc-based MOFs play a pivotal role in the process of bone regeneration, owing to their status as a fundamental trace element critical for immune function, cellular proliferation, and skeletal development. These attributes render zinc a valuable constituent in biomaterials designed for orthopedic and dental applications.^57^ Zinc and its alloys exhibit mechanical properties comparable to mammalian bone, positioning them as promising candidates for load-bearing scaffolds, largely due to the physiological functions of Zn^2+^ ions.^58–60^ Notably, approximately 90% of the body’s zinc is localized within bone tissues and muscle.^61^ While zinc toxicity is uncommon, zinc deficiency is relatively prevalent and can negatively impact growth, neural development, and immune responses; conversely, excessive zinc intake may provoke copper deficiency. The integral role of zinc in bone regeneration highlights its importance in enhancing strategies for orthopedic biomaterials and bone tissue engineering.^62,63^

Zn^2+^ ions exert concentration-dependent effects on bone physiology and regenerative processes. Concentrations ranging from 7 and 20 nM have been demonstrated to stimulate ALP activity, a critical enzyme for bone mineralization, while beneficial effects on osteoblast function persist at concentrations up to 50 nM.^64,65^ Moreover, Zn^2+^ has been reported to inhibit osteoclastogenesis, indicating its therapeutic potential in managing osteoporosis.^57,66^ In scaffold-based bone tissue engineering, Zn^2+^ modulates β-catenin signaling pathways, including activation of Wnt pathway components such as Axin2 and LRP5, alongside upregulation of the osteoclast-related gene RANKL, underscoring its regulatory influence on osteoclast differentiation.^67^ Supplementation with Zn^2+^ has also been shown to enhance extracellular matrix mineralization in human mesenchymal stem cell cultures, promoting the expression of osteogenic markers including osteopontin and ALP.^56,68,69^

Additionally, Zn^2+^ exhibits concentration-dependent cytotoxic effects on smooth muscle cells at micromolar concentrations between 80 and 120 μM.^70^ Investigations into Zn^2+^-modified titanium coatings suggest a preference for Zn^2+^ ions at the biomaterial interface, resulting in improved cellular responses.^71^ Zinc supplementation further facilitates collagen synthesis and mineral deposition in osteoblast-like cells, while simultaneously inhibiting osteoclastogenesis and promoting osteoblast differentiation and activity, thereby holding promising implications for bone tissue engineering and regenerative medicine.^57,65^

Elevated serum zinc levels have been correlated with improved bone health outcomes, including increased BMD and a reduced risk of fractures.^72^ Clinical studies, such as one involving patients with thalassemia, reported significant enhancements in both bone mineral content (BMC) and BMD following an 18-month regimen of Zn^2+^ supplementation (25 mg/day) relative to placebo controls.^73^ In bone regeneration contexts, zinc phosphate-loaded barrier membranes display notable antimicrobial properties, effectively preventing bacterial colonization and minimizing infection risks.^74^ Furthermore, in grafting applications, cross-linked gelatin membranes embedded with powder of zinc hydroxyapatite have demonstrated superior bone defect filling (approximately 80%) in rat calvarial defect models compared to collagen membranes and untreated controls.^75,76^ Recent advancements have highlighted the antibacterial efficacy, excellent biocompatibility, and osteostimulatory effects of nanocomposites composed of carboxylated graphene oxide sheets decorated with zinc oxide nanoparticles, further supporting zinc’s utility in nanoparticle-based tissue engineering formulations.^77,78^

Zinc ions that are released from biomaterials composed of zinc-doped tricalcium phosphate significantly influence bone cell activity and bone formation mechanisms. These ions have been observed to enhance TRAP and ALP activities in human bone marrow-derived mesenchymal stem cells, while also modulating the formation and function of multinucleated giant cells in RAW264.7 macrophage cultures.^57,75,79^ Canine ectopic implantation studies have demonstrated that de novo bone formation occurs exclusively with zinc incorporation in TCPs, underscoring zinc’s essential role in bone regeneration.^79^ Furthermore, zinc has shown considerable promise in the development of implant coatings to improve osseointegration. Zinc-loaded titanium oxide coatings upregulate the expression of osteogenic genes and promote early stage new bone formation in comparison to coatings lacking zinc.^80^ Similarly, zinc-modified calcium silicate coatings have been reported to enhance osteogenic differentiation and mineralized matrix formation around titanium implants, particularly in osteopenic rabbit models. Molecular investigations suggest that zinc exerts regulatory control over the transforming growth factor-beta (TGF-β)/Smad signaling pathway, which is crucial for osteoblastogenesis.^81^ Collectively, these findings emphasize zinc’s multifaceted potential to facilitate implant integration, accelerate bone regeneration, and inhibit biofilm formation, establishing it as a significant contender in the fields of tissue engineering and regenerative therapies.^53,57,75^

Various MOF materials have been extensively investigated for bone-related treatments, ranging from basic infections to complex bone cancers. Among these MOFs, zinc-based frameworks, particularly ZIF-8, have garnered considerable attention over the past decade. This is largely attributed to their distinctive morphological features, multifunctionality, corrosion resistance, and notable biological properties, including bioactivity, biocompatibility, osteogenic potential, and angiogenic capabilities.^82^ A representative example is the multifunctional hydrogel composed of catechol-modified chitosan (CA-CS) integrated with ZIF-8 nanoparticles (ZIF-8 NPs), known as CA-CS/Z hydrogel, which was fabricated through the homogeneous blending of two presynthesized solutions and characterized comprehensively by Liu et al. (Figure 3).

**Figure 3:**
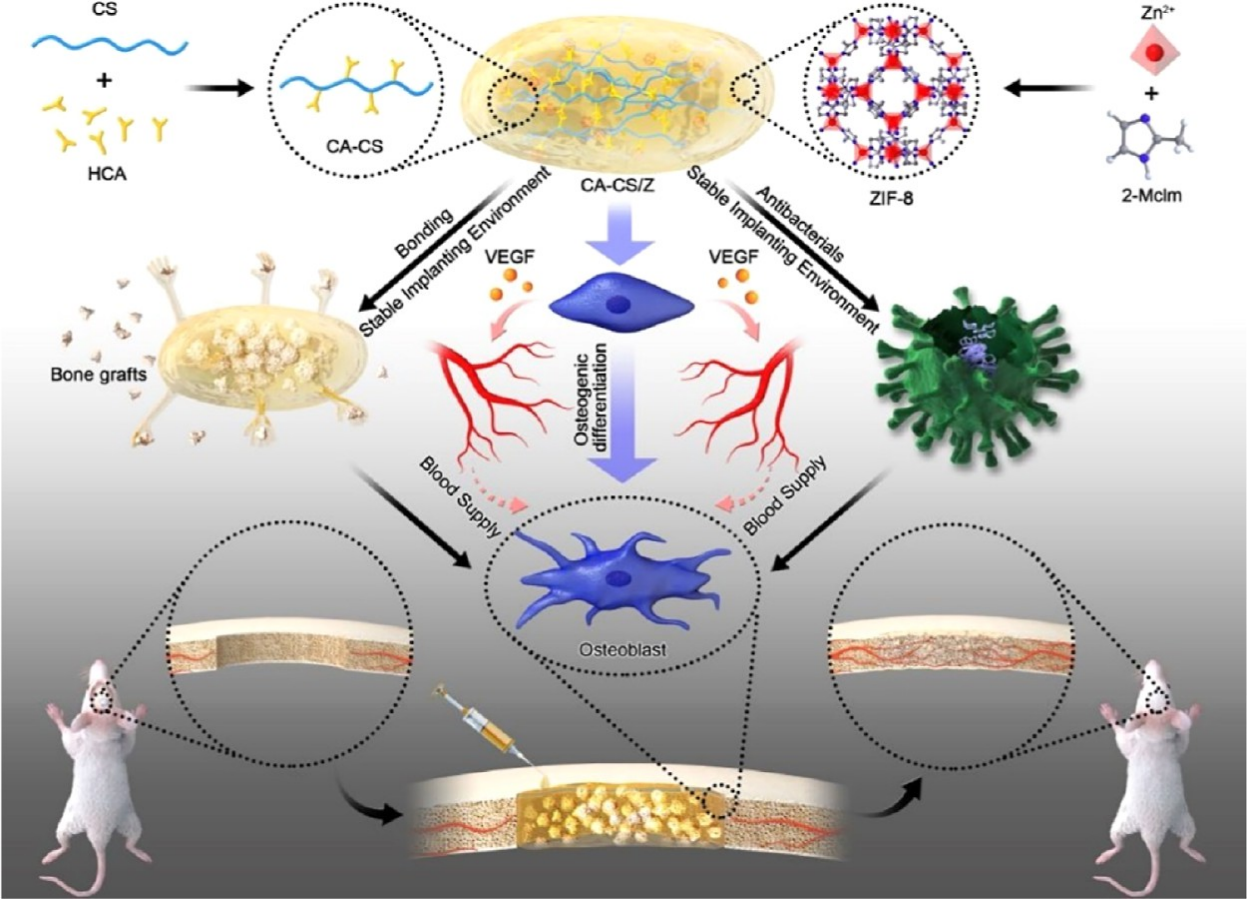
Schematic illustration of the design and application of the multifunctional bone-adhesive hydrogel CA-CS/Z^83^. Reproduced from ref 83. Copyright 2020 American Chemical Society.

SEM images of the lyophilized hydrogels revealed uniform morphological dimensions regardless of variations in composition, suggesting that the inclusion of ZIF-8 NPs does not adversely affect the structural integrity of the hydrogel matrix (Figure 4B). Furthermore, the pore size of the hydrogels was consistently below 30 μm and notably decreased with increasing ZIF-8 content, which may influence cellular infiltration and nutrient transport (Figure 4C). ICP-AES analysis confirmed a sustained yet gradually diminishing release of Zn^2+^ ions over time, with a marked decrease observed after the initial day.

**Figure 4:**
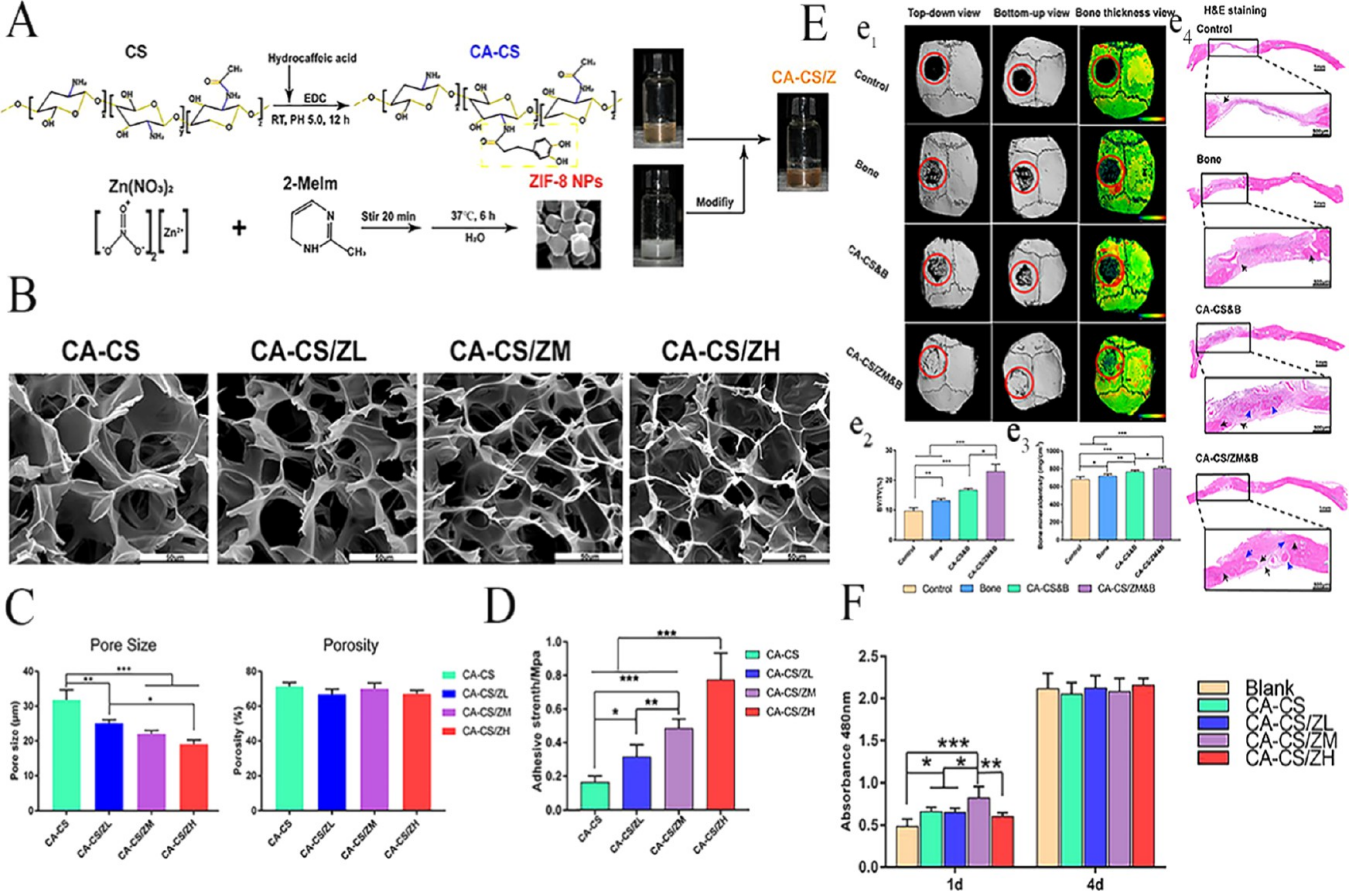
CA-CS/Z (zeolitic imidazolate framework-8 nanoparticles (ZIF-8 NPs) modified catechol-chitosan (CA-CS) multipurpose hydrogels. (A) Fabrication of CA-CS/Z. (B) SEM images. (C) The pore size and porosity. (D) Tensile adhesive strength. (E) Micro-CT assessment and hematoxylin–eosin (H&E) staining. (e_1_) Micro-CT reconstructions of the rat skulls. (e_2_) BV in the cranial defect. (e_3_) BMD in the cranial defect. (e_4_) H&E staining for regenerated bone tissues. (F) CCK-8 assay results of rBMSCs cultured in conjunction with different hydrogels.^83^ Reproduced from ref 83. Copyright 2020 American Chemical Society.

The biological efficacy of CA-CS/Z hydrogels was assessed by using rat bone marrow mesenchymal stem cells (in vitro) and rat calvarial defect models (in vivo). These studies demonstrated that the hydrogel significantly enhances cell adhesion while mitigating cytotoxic effects. Importantly, the hydrogel promoted angiogenesis and osteogenesis, as evidenced by upregulation of osteogenic genes and increased secretion of osteoblast-associated proteins. Additionally, the material facilitated revascularization concurrent with new bone formation (Figure 4D–F).^83^ These findings collectively underscore the hydrogel’s potential as a versatile scaffold for bone regeneration, combining structural support with bioactive ion release to regulate cellular activities favorable for tissue regeneration.

Furthermore, zinc-based MOFs have shown considerable potential in improving the process of bone repair through the controlled release of therapeutic ions and drugs, alongside modulation of the ionic microenvironment. A pertinent example is the zeolitic imidazolate framework-8-modified implant (Z-AHT), synthesized by Zhang et al., which is further functionalized with dimethyloxalylglycine (DMOG) to generate D-AHT. The Z-AHT implant was prepared via solvothermal synthesis of a ZIF-8 coating on alkali heat-treated titanium (AHT), while D-AHT was subsequently obtained by immersing Z-AHT in a solution of deionized water containing DMOG.

SEM analysis revealed that the crystalline structures on the surface of Z-AHT exhibited a morphology of a rhombic dodecahedron with an average size of approximately 300 nm. In contrast, the drug-loaded D-AHT displayed smaller, rounded crystals, indicative of structural modification due to drug incorporation (Figure 5A). This distinctive surface architecture provides advantages for drug loading and sustained release, as confirmed by release profiles depicted in Figure 5B,D. Moreover, hydrolysis of the hydroxyl layer on the alkali titanate surfaces imparted excellent wettability to the otherwise hydrophobic titanium substrates, facilitating robust implant integration (Figure 5C).

**Figure 5:**
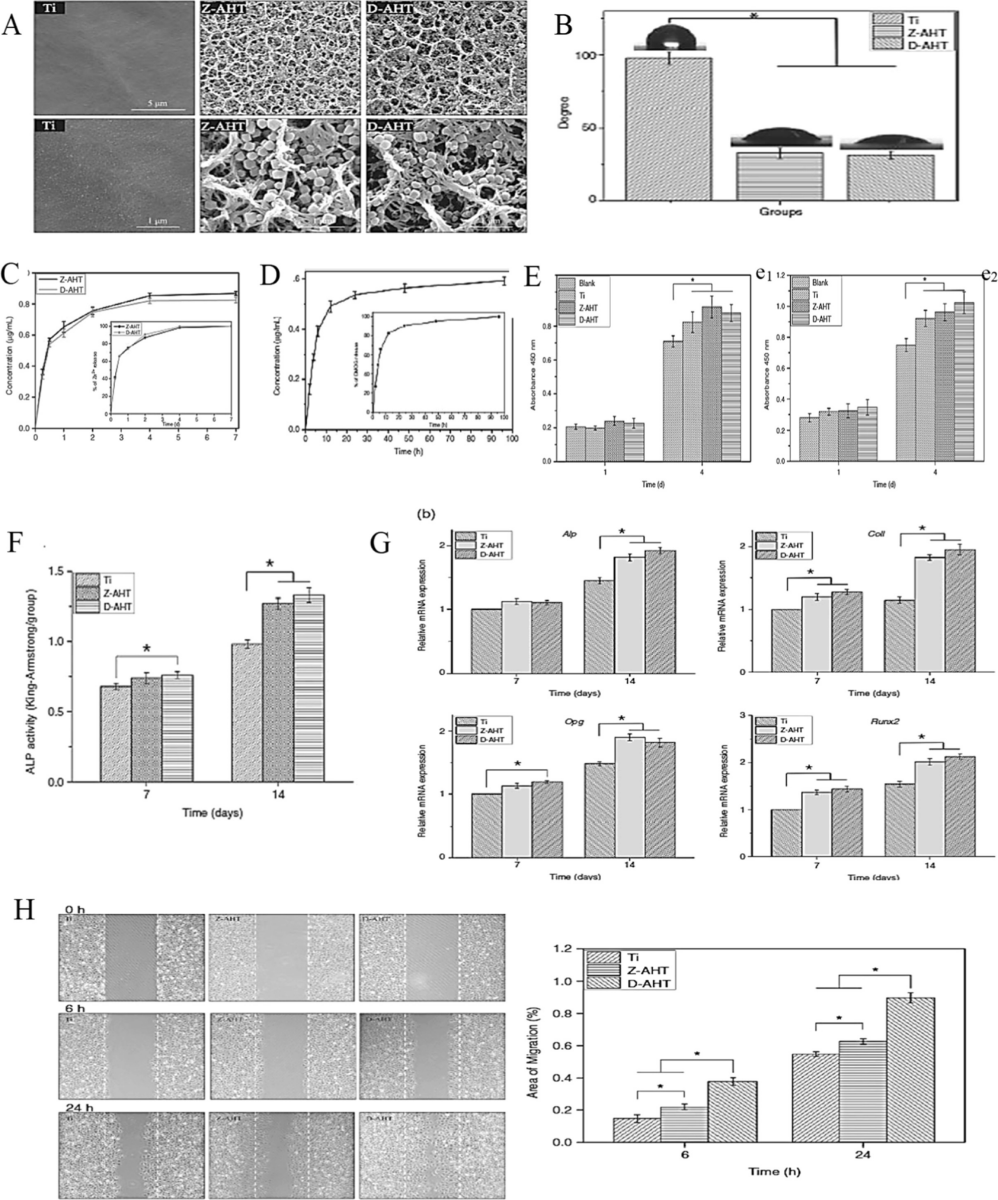
Good drug loading and release by zeolitic imidazolate framework-8-modified implants (Z-AHT) loaded by dimethyloxalylglycine (D-AHT). (A) SEM images. (B) The release of DMOG from the D-AHT. (C) Contact angles. (D) Kinetics of zinc ion release from Z-AHT and D-AHT in PBS. (E) Cell proliferation assessed using a CCK-8 assay (e_1_) MC3T3-E1 cells and (e_2_) HUVEC. (F) Quantitative ALP activity. (G) Expression of genes associated with osteogenesis in MC3T3-E1 cells. (H) Cell migration assay.^84^ Reproduced with permission from ref 84. Copyright 2019 Sage Publications.

Cell adhesion is a critical parameter influencing the subsequent proliferation and viability. The modified implants exhibited enhanced wettability that supported proliferation and adhesion of MC3T3-E1 and HUVECs, thereby promoting cellular viability (Figure 5E). To evaluate osteogenic activity, the expression of osteogenesis-related genes, including Alp, Col1, Opg, and Runx2, and ALP activity, was assessed. Both D-AHT and Z-AHT implants induced significantly higher ALP activity in MC3T3-E1 cells relative to unmodified titanium controls, accompanied by notable upregulation of osteogenic gene expression in cells cultured on Z-AHT (Figure 5F,G). Furthermore, enhanced migration of HUVECs was observed upon release of Zn^2+^ ions and DMOG from the implants, underscoring the angiogenic potential of D-AHT (Figure 5H).^84^ The following table demonstrates the recent Zn-based MOFs studied in bone disease treatment (Table 1).

**Table 1:** Zn-Based MOFs Studied in Bone Disease Treatment

MOF diversity	characteristics of MOFs	type of function	application	in vivo test	cell viability	cell name	method of synthesis	refs
Bio-MOF-1on Mg alloy	thickness: 190 μm	implant	bioactive, corrosion resistance	not mentioned	80%	L929 mouse fibroblast cells	hydrothermal synthesis method	^85^
ZIF-8 MOF/chitosan on Mg alloy	particle size: 65 ± 5 nm, thickness ≈ 22 nm, specific surface area: 1789 m2 g–1, average pore diameter: 2.9 nm, total pore volume: 1.29 cm^3^/g, average diameter of MOF/chitosan composite: 200 nm	implant	biocompatible, bioactive, biodegradability	not mentioned	70%	MG-63	electrospinning synthesis method	^86^
PLLA/ZIF-8@ PDA-HA	size ≈ 266.5 nm	scaffold	biocompatible, bioactive	not mentioned	Abs = 0.8	MG-63	3D printing technology	^87^
PCL/DCPD/nano-ZIF-8	size: 445 ± 34 nm	scaffold	osteogenesis, biocompatible	rabbit critical calvarial defect model	Abs = 1.8	RBMSCs	3D printing technology	^88^
CA-CS/Z	size< 30 μm	hydrogel	osteogenesis, angiogenesis, antibacterial	rat calvarial defect model	not mentioned	RBMSCs	homogenous mixing of ZIF-8 NPs and CA-CS	^83^
PCL/BMP@ZIF-8	size of ZIF-8:83 nm ± 18, size of BMP@ZIF-8:68 nm ± 15	implant	drug delivery, osteogenesis	rat cranial bone defect	Abs = 1.2 after 21 days	mouse preosteoblasts (MC3T3-E1 cells, RCB1126, Riken)	electrospinning synthesis method	^89^
SF-DEX@ZIF-8-Ti	surface area of ZIF-8:1392 m^2^ g^–1^, surface area of DEX@ZIF-8:1244 m^2^ g^–1^, size: both of them are about 60 nm	implant	drug delivery, osteogenesis	not mentioned	nontoxic	MC3T3-E1 cells	conventional synthesis of MOF	^90^
Z-AHT loaded with DMOG (D-AHT)	size of ZIF-8 on Z-AHT: 300 nm, contact angles of Z-AHT: 32.8 ± 3.7 and D-AHT:31.6 ± 2.6°	implant	ion delivery, osteogenesis, angiogenesis	not mentioned	Abs = 0.9 Abs = 1.10	MC3T3-E1 cells HUVECs	solvothermal synthesis method	^84^
PG/Aln-ZIF-8	size of ZIF-8 in PG/ZIF-8:120 ± 25 nm, Size of ZIF-8 in PG/Aln- ZIF-8:130 ± 30 nm, surface roughness of LBL-MOF/Ral: 122 ± 24 nm	implant	drug and ion delivery, antiosteoporosis, osteoinductive, antibacterial	rat circular bone defects	nontoxic	MC3T3-E1 cells RAW264.7 cells	electrospinning synthesis method and a simple solution-phase synthesis	^91^
LBL-MOF/Ral	particle size of LBL-MOF group: about 120 nm, particle size of LBL-MOF/Ral group: about 65 nm	implant	osteogenesis	Femurs of osteoporotic rat cells	Abs = 1.3 after 7 days	MC3T3-E1 cells	conventional synthesis of MOF on LBL-Zn sample	^92^
PLLA@Zn–Cu MOF	pore size: 10.9 nm, surface area: 6 m2 g–1, crystallite size of MOF: 60 nm	scaffold	osteogenesis	not mentioned	not mentioned	HADMSCs	MOFs coating on electrospinning PLA scaffold	^93^
PLA ZIF-11; PLA HKUST-1	HKUST-1: Zeta potential: 54.23 ± 0.04, particle Size distribution: 1480 nm, hydrophobicity: 91.4 ± 1.6°. ZIF-11: Zeta potential: –6.33 ± 0.02, particle Size distribution: 1990 nm, hydrophobicity: 102.3 ± 2.5° Both size distribution: 1–2 μm, hydrophobicity of pure PLA: 108.1 ± 1.4°	scaffold	ion delivery, tenogenesis, osteogenesis, angiogenesis	rat RCT model	Abs = 2.1 after 5 days	RAMSC	electrospinning synthesis method	^94^
ZIF-8 (nano and micro)	crystal size of microZIF-8 > 10 μm, crystal size of nano-ZIF8:200–300 nm, coating thickness: 10 μm	implant	antibacterial, osteogenesis, biocompatible	not mentioned	not mentioned	MG-63 cells	Nano-ZIF-8: hydrothermal synthesis method, MicoZF-8: solvothermal synthesis method	^95^
ZIF-8@Levo/LBL	Zeta potential of ZIF-8: + 22.6, Zeta potential of ZIF-8@Levo: + 8.8 Mv, Crystal size of ZIF-8:136 ± 28 nm, crystal size of ZIF-8@Levo: 189 ± 35 nm	implant	antibacterial, osteogenesis	Femurs of rats	Abs = 1.4 after7 days	Rob cells	layer-by-layer (LbL) deposition	^96^
PDGF@ZIF-8-PDA@COL/PLGA-TCP	size of ZIF-8-PDA: 226.2 ± 5.3 nm	implant	antibacterial, osteogenesis, biocompatible	rat cranial bone defect	not mentioned	RMSCs	perfusion of PDGF@ZIF-8-PDA@COL hydrogels into PLGA-TCP scaffolds after layer-by-layer deposition of PDGF@ZIF-8-PDA@COL	^97^
FA/MOF/DOX	size of MOF/DOX particles: 95.8 ± 4.7 nm, size of FA/MOF/DOX: 106.3 ± 3.9 nm	nanoparticle	antiosteosarcoma	MG-63 xenografted nude mice	not mentioned	MG-63	DOX loading on encapsulated MOF	^98^
cisplatin or BMP-2 encapsulated pZIF-8 nano-MOFs	pZIF-8 nano-MOF size≈ 50 nm, pore size ≈ 1 nm, Zeta potential of pZIF-8 nano-MOFs: −19.3 ± 0.4 mV, Surface area of ZIF-8:1400 cm^2^/g, Surface area of pZIF-8 nano-MOF: 1380 cm^2^/g	scaffold	osteogenesis, anticancer	Rabbit femoral defect model	Abs = 1.4 after 7 days	BMSCs	conventional synthesis method	^99^
DZIF@PGel	pore size of DZIF@PGel: 200–400 μm	hydrogel	antibacterial, osteogenesis, anti-inflammatory	rat model	not toxic	HGFs and OB cells	UV light irradiation of DZIF@PGel hydrogel after step-by-step synthesis	^100^
PCL/Col/ZIF-8	crystal size of ZIF-8≈ 300 nm	composite membrane	tendon and bone healing, osteogenesis, angiogenesis	CAM, Rat calvarial bone defect model	not toxic	RBMSCs, L929 mouse fibroblast cells	hydrothermal synthesis method	^101^
Bio-MOF-1@AHT	Surface wettability of uncoated Ti: 119.2°, of Bio-MOF-1@AHT: 90.5–22.9°, Crystal size: 300 to 500 nm	implant	osteogenesis	tibia defect rabbit model	not toxic	BMSCs	high temperature synthesis of MOF on solvothermal prepared AHT	^102^
PCL/LIG/ZIF-8	contact angle of PCL/LIG/ZIF-8:24.2°	nanofibers	osteogenesis, antibacterial	not mentioned	not toxic	RBMSCs HBMSCs	conventional synthesis of ZIF-8 on electrospun PCL/lignin (PCL/LIG) nanofibers	^103^
ZIF-8@VAN@BG	not mentioned	scaffold	osteogenesis, antibacterial	not mentioned	not toxic	RBMSCs	in situ deposition of ZIF-8@VAN on the BG scaffolds	^104^
ZIFMPCs	not mentioned	bone cement	osteogenesis, biocompatibility	not mentioned	not mentioned	MBMSCs	step-by-step synthesis of ZIF-8 and ZIFMPCs	^105^
DEX@Zn–Mg-MOF74	crystal size of Zn–Mg-MOF 74/PDA: 2–3 μm, WCAs of the PEEK, PEEK–PDA, PEEK-74, and PEEK–DEX: 80, 45, 0, and 0°	implant	osteogenesis, biocompatibility, angiogenesis, antibacterial, Drug and ion delivery	rat femur drilling model	not mentioned	RBMSCs	loading by drop on the hydrothermally synthesized Zn–Mg-MOF 74/PDA	^106^
Nano-ZIF-8	particle size: 200 nm	implant	Osteogenesis, Chondrogenesis	SD rats	not toxic	RBMSCs	one-pot synthesis method	^107^
PP/PDA/ZIF-8	not mentioned	implant (membrane)	Osteogenesis	not mentioned	Abs = 0.7 after 7 days	HDPSCs	step-by-step preparation of the membrane	^108^
miR@ZIF-8	particle size of ZIF-8:236.6 ± 47.3 nm, particle size of miR@ZIF-8 ≈ 242 nm	nanocomposites (carrier)	Angiogenesis, Osteogenesis, Biomolecule delivery	CAM, a critical-sized cranial defect model of rats	not toxic	BMSCs, HUVEC cells	one-pot synthesis method	^109^

Reviewing the data presented in the table demonstrates that it is evident that Zn-MOFs exhibit substantial variability in their physical characteristics, functional attributes, and biological efficacy in bone disease treatment. Zn-MOFs with smaller particle sizes and higher specific surface areas, such as the ZIF-8 MOF/chitosan composite and SF-DEX@ZIF-8-Ti, generally demonstrate superior capabilities for cellular interaction and therapeutic agent delivery. These attributes facilitate an enhanced osteogenic potential and sustained bioactivity. Conversely, Zn-MOFs with larger particle sizes or those lacking multifunctionality such as certain bulkier Bio-MOF-1 on a Mg alloy may exhibit comparatively reduced bioactivity and slower drug release profiles, which could limit their therapeutic efficacy.

Furthermore, synthesis techniques such as electrospinning and 3D printing enable the fabrication of scaffolds (PLLA/ZIF-8@PDA-HA and PCL/DCPD/nano-ZIF-8) that better mimic natural bone architecture, thereby improving cell adhesion, proliferation, and tissue integration. Moreover, composite hydrogels (CA-CS/Z) and multifunctional implants (PCL/BMP@ZIF-8) combine osteogenesis with antibacterial and angiogenic effects, which are crucial for complex bone defects.

Additionally, materials that incorporate bioactive molecules such as BMP and dexamethasone (PCL/BMP@ZIF-8 and SF-DEX@ZIF-8-Ti) provide added therapeutic advantages through controlled drug delivery, setting them apart from bare MOF structures. In vivo validation remains a critical aspect; Zn-MOFs tested in animal models for bone regeneration confirm their clinical potential, while those lacking such assessments require further investigation. Most Zn-MOFs also show excellent biocompatibility with relevant bone-related cell types, maintaining high cell viability and supporting regenerative processes.

In summary, Zn-MOFs synthesized via advanced methodologies and engineered for multifunctionality consistently outperform simpler or untested formulations. Future developments should focus on optimizing parameters such as particle size, surface area, bioactivity, drug loading, and scaffold architecture to maximize therapeutic outcomes. The integration of Zn-MOFs with growth factors or antibiotics, combined with scalable production methods, promises to advance their translational potential as effective biomaterials for bone tissue engineering and regenerative medicine.

#### 4.1.2 The Role of Mg^2+^ and Mg-Based MOFs in Bone Regeneration

As a fundamental component of the human body, magnesium (Mg^2+^) plays several vital roles for life processes, for instance, its participation in enzymatic processes, antioxidative and antiapoptotic effects, and association with osteogenesis and angiogenesis.^110^ Magnesium is a vital ion necessary for the human body, acting as a vital transporter in the synthesis of the bone matrix, thereby supporting skeletal strength and integrity.^75^ Additionally, magnesium facilitates the growth of osteoblasts, proliferation, and adhesion, which are crucial steps preceding the mineralization of bone tissue. Moreover, magnesium exerts anti-inflammatory effects by reducing the levels of pro-inflammatory mediators and simultaneously increasing the expression of anti-inflammatory cytokines.^56,111,112^

Mg-based alloys are categorized as third-generation biomaterials and hold exceptional promise for bone defect repair. These biodegradable materials represent a pioneering advancement in orthopedic implants, designed to closely replicate the mechanical characteristics of natural bone tissue.^112^ Due to their inherent biodegradability, biocompatibility, bioactivity, and biotolerance, Mg alloys offer a compelling alternative to conventional permanent implants.^85^ Serving as temporary frameworks within biological environments, these alloys gradually degrade, aligning with the dynamic demands of tissue healing and regeneration.^75,113^

In comparison to other biomaterials such as titanium alloys and stainless steel, Mg alloys present unique advantages.^113^ The density and elastic modulus of these materials closely mirror those found in natural bone, thereby reducing stress shielding effects. Furthermore, their biodegradability eliminates the necessity for secondary removal surgeries, which is a limitation of nondegradable implants. Nonetheless, the application of magnesium alloys in clinical settings is presently limited because of their swift corrosion, which can compromise mechanical stability before complete tissue healing.^114^ Despite excellent biocompatibility, overcoming this corrosion challenge has led to various approaches, including careful selection of alloying elements, surface modification techniques, and coating strategies. Notably, surface coatings composed of bioactive ceramics or biodegradable polymers have shown promising potential in delaying biodegradation, thereby enhancing clinical applicability.^115,116^

In the design of bone repair implants, magnesium alloys require properties beyond corrosion resistance, including bioactivity, antibacterial effects, and hydrophilicity. Coatings that combine protective functionality with the ability to promote mineralization are particularly attractive, as they can provide sustained protection while encouraging the formation of mineral layers conducive to bone healing. Magnesium alloys also benefit from their lightweight nature, density akin to natural bone,^75,115,116^ and superior strength-to-weight ratio,^117^ reinforcing their status as promising candidates for orthopedic applications. Nevertheless, comprehensive protective interventions remain critical to ensuring their long-term success in physiological environments.

In addition to Zn-based MOFs, other metallic elements can be utilized in the fabrication of biomaterials aimed at bone regeneration. Magnesium-based MOFs serve as a notable example, given that Mg^2+^ ions play a biotic role in the synthesis of bone matrix. This is partly due to their density, which closely approximates that of natural bone (natural bone density: 1.80–2.00 g/cm^3^ and Mg^2+^: 1.74–2.00 g/cm^3^).^111,113^

An illustrative case is the PLGA/Exosome-Mg-gallic acid (Mg-GA) MOF composite developed by Kang et al. for the purpose of bone defect repair. In their research, the MOFs were synthesized through a hydrothermal method, subsequently leading to the creation of PLGA/Mg-GA MOF composite scaffolds using electrospinning technology with subsequent functionalization through exosome incorporation. Two critical parameters, pore size and surface area, were identified as essential factors in designing effective bone substitutes to facilitate osteogenesis and neovascularization. Observations from digital photography and SEM confirmed that all scaffold groups exhibited a favorable fibrous morphology. BET analysis revealed that the composite scaffold’s surface area diminished from 229.4 m^2^/g to 154.5 m^2^/g upon exosome addition, indicating strong interactions between the MOF surface and exosomes. Electrostatic interactions were further evidenced by a shift in the zeta potential from negative to positive following exosome binding, reinforcing the presence of this interaction.

A critical requisite for in vivo tissue engineering is creating an environment conducive to the adhesion and proliferation of hBMSCs. Accordingly, the study employed DAPI/FITC-phalloidin fluorescence staining to evaluate the proliferation on the scaffolds and cell adhesion. Both PLGA/Mg-GA_1_ and PLGA/Mg-GA_2_ surfaces were well covered by hBMSCs, with cells displaying polygonal morphology and visible intercellular filaments. Cytotoxicity evaluation via the CCK-8 assay demonstrated that the scaffolds exerted no toxic effects on cells after 3 days of culture. Furthermore, osteogenic differentiation markers, ALP, Runx2, and OCN, were significantly upregulated after 14 days of hBMSC culture on the scaffolds. The scaffolds also promoted VEGF expression in hBMSCs, enhancing the migration and tube formation in HUVECs.

The osteogenic, angiogenic, and anti-inflammatory efficacy of these scaffolds was further validated using a rat calvarial defect model, with micro-CT imaging illustrating their potent bone regenerative capacity. Kang et al. attributed these positive outcomes to the sustained release of Mg^2+^ ions and GA, the extensive surface area of the Mg-GA MOF, and its unique nanostructure, all of which synergistically contributed to the enhanced osteogenic performance observed.^118^

Despite the notable advantages of Mg-based MOFs, their clinical application is hindered by poor corrosion resistance in physiological fluids.^113^ To address this limitation, various hybrid metal coatings have been developed and investigated. For instance, Shen et al. made up a hybrid Mg/Zn-MOF74 coating on an AT substrate using a solvothermal synthesis approach. Beyond the well-documented biological benefits of Zn^2+^ ions, the incorporation of zinc was anticipated to enhance the water stability of Mg-MOF74, attributable to the differing reduction potentials of Zn^2+^ (−0.76 V) and Mg^2+^ (−2.37 V).

SEM and EDS analyses demonstrated that reducing the Mg^2+^ content while increasing Zn^2+^ concentration resulted in smaller surface particle diameters and decreased coating thicknesses. Evaluations of wettability, performed via measurements of the water contact angle, indicated that the AT-Mg/Zn-coated surfaces exhibited contact angles near 9°, indicating they were marginally less hydrophobic than AT alone (approximately 5.2 ± 1.2°) but significantly more hydrophilic than native titanium surfaces, which displayed contact angles around 62.7 ± 3.2°.

The deterioration behavior of the Mg/Zn-MOF74 coating is pH-dependent, and since bacterial proliferation often induces an acidic microenvironment, the MOF-coated implant consequently possesses effective antibacterial properties. Although the MOF74-coated implants initially exhibited cytotoxic effects on osteoblasts during early stages of cell culture, a three-day presoaking period in PBS markedly enhanced both osteoblast proliferation and osteogenic differentiation. Additionally, the MOF74-modified implants demonstrated anti-inflammatory and antibacterial effects during the initial stages postimplantation. This multifunctionality is primarily due to the generation of an alkaline microenvironment, driven by the controlled release of organic ligands and metal ions, resulting in a pH of approximately 8.0 to 8.5. Previous studies have indicated that such a mildly alkaline milieu is conducive to osteoblast differentiation and proliferation while simultaneously reducing the risk of infection.^119^

Moreover, Mg^2+^ and Zn^2+^ ions exert a significant influence on the anti-inflammatory and osteogenic activities of MOF74-modified materials. Specifically, Mg^2+^ concentrations between 0.5 and 2.0 mM have been shown to upregulate mineralization in human osteoblasts, osteocalcin expression, and ALP activity, whereas concentrations exceeding 4.0 mM have opposite, detrimental effects.^120^ Similarly, Zn^2+^ ions possess both pro-osteogenic and anti-inflammatory properties; Kim et al. reported that excessive Zn^2+^ levels, with an EC50 range of approximately 5–44 ppm, exert potent anti-inflammatory effects.^121^ These findings suggest that MOF74-modified samples hold promising potential for advancement in antibacterial, anti-inflammatory, and osteogenesis-promoting applications in vivo.^122^

Bone defect treatment in diabetic patients is one of the significant challenges in the clinic. Accordingly, Hua et al. synthesized the magnesium/emodin-based metal–organic framework (MgEm MOF). The schematic illustration is demonstrated in Figure 6. That was synthesized via a hydrothermal method by dissolving emodin and magnesium chloride in deionized water, adjusting the pH to 8.0 using sodium hydroxide, and heating at 80 °C for 24 h. The resulting precipitate was centrifuged, ultrasonicated, washed with water, and lyophilized to yield tawny MgEm MOF powder, which was stored at 4 °C.

**Figure 6:**
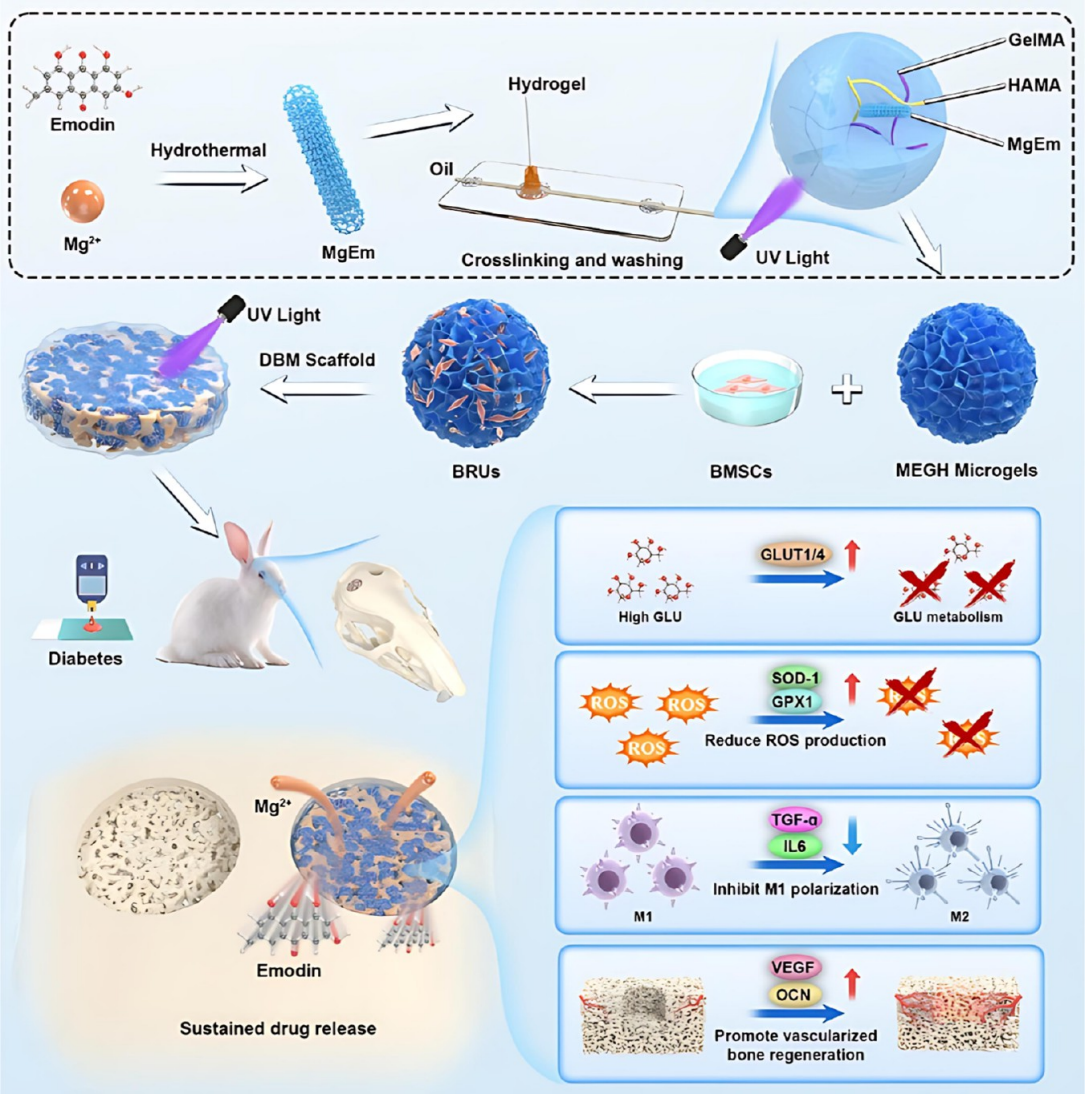
Schematic representation of the repair process of diabetic rabbit skull bone defects using BMSCs@MEGH-D scaffolds, along with an explanation of the mechanisms involved in reversing the pathological microenvironment caused by diabetes.^123^ Reproduced from ref 123. Available under a CC-BY 4.0 license. Copyright 2024 Wang et al.

For GelMA/HAMA-encapsulated microgel (MEGH) fabrication, GelMA and HAMA were dispersed in PBS with a MgEm MOF and a photoinitiator, forming the aqueous phase. This was combined with a paraffin oil-based continuous phase in a microfluidic chip, where shear forces and UV cross-linking (365 nm, 20 mW/cm^2^) produced stable microgel microspheres (∼300 μm diameter). The MgEm MOF nanorods and MEGH microgels represent a promising multifunctional platform for diabetic bone regeneration, as demonstrated by comprehensive characterization.

SEM analysis revealed uniform rod-like MgEm nanorods (∼100 nm diameter, ∼700 nm length) with homogeneous elemental distribution (62.40% C, 34.44% O, 3.16% Mg via EDS) and high crystallinity (XRD), distinct from precursors. MEGH microgels, fabricated via microfluidics, exhibited monodispersity (∼300 μm diameter) and successful MgEm encapsulation, confirmed by SEM and optical imaging. Both systems showed excellent cytocompatibility with BMSCs at concentrations up to 10 μg/mL (MgEm) and 1 mg/mL (MEGH extracts) after 48 h. Release kinetics indicated a biphasic profile for emodin (73% loading) and Mg^2+^ (11.65% loading), with MEGH providing slower, sustained release over 90 days compared with bare MgEm, enhancing therapeutic potential for long-term applications.

Biological evaluations underscored the efficacy of MEGH microgels and MgEm nanorods in addressing the complex diabetic bone defect microenvironment. In vitro, MEGH supported robust BMSC viability (>95%), adhesion, spreading, and proliferation over 14 days, enabling bone regenerative unit formation. MgEm nanorods reduced glucose levels by upregulating GLUT1 and GLUT4, mitigated oxidative stress (modulating SOD-1, GPX1, and NRF2), suppressed inflammation by reducing CCR7, TGF-α, and IL-6 in macrophages, and enhanced angiogenesis through HUVEC migration and VEGF upregulation. By upregulating OCN and mRNA, osteogenesis was enhanced.

In vivo, BMSCs@MEGH-D scaffolds in nude mice and diabetic rabbit skull defect models significantly improved vascularized bone regeneration by 8 and 12 weeks, respectively, with enhanced bone volume, surface area, mechanical properties and expression of OCN and CD31, while reducing inflammation. Transcriptomic analysis confirmed cooperative control of glucose metabolism, oxygen bioprocesses, inflammation, and the pathways of osteogenesis and angiogenesis, positioning this system as a novel strategy for diabetic bone repair.^123^ A summary of the latest research on Mg-based MOFs in the treatment of bone diseases is provided in Table 2.

**Table 2:** Mg-Based MOFs Studied in Bone Disease Treatment

MOF diversity	characteristics of MOFs	type of function	application	in vivo test	cell viability	cell name	method of synthesis	refs
PT/SF/ICA@MOF	pore size of MOF: 6–11 nm, pore size of ICA@MOF: 1.5–5 nm	scaffold	Biocompatible, osteogenesis, drug delivery, osteoporotic integration, anti-inflammatory	C57BL/6 mice	not mentioned	Raw264.7	ICA@MOFs and SF solution injected into 3D printed PT scaffold	^124^
Mg-MOF-74/MgF_2_	WCA≈ 0°	implant	anticorrosion, hydrophilic coating for Mg alloys	not mentioned	not mentioned	not mentioned	Pretreatment of hydrofluoric acid and in situ hydrothermal synthesis methods	^114^
PLGA/Exo-Mg-GA MOF	zeta potential of Mg-GA MOF: + 10.9 ± 0.5 mV surface area of Mg-GA MOF: 229.4 m^2^/g, surface area of Exo-Mg-GA MOF: 154.5 m^2^/g	scaffold	anti-inflammatory, osteogenesis, angiogenesis, biocompatible	rat calvarial defect model	not mentioned	HBMSCs, HUVECs	electrospinning method	^118^
MOF@CaP	surface area of MOF@CaP: 228.7 m^2^/g; pore size of MOF@CaP: 8.1 nm; size of MOF@CaP: 100 nm	scaffold	Anti-inflammatory, ion and drug delivery, osteogenesis, angiogenesis, biodegradable	rat defected model	not mentioned	RAW264.7, HUVEC	(CaP-modified MOF)/collagen scaffold	^55^
Sr-HA-MOF74	contact angles: about 10°	filler	antitumor, antibacteria, ion delivery, bioactivity, biocompatibility, osteogenesis	not mentioned	not mentioned	Saos-2 cells, primary osteoblasts	solvothermal synthesis method	^125^
Ket@Mg-MOF-74	size: micrometer	carrier	drug delivery, ion delivery osteoporotic bone pain treatment, anti-inflammatory	not mentioned	Abs >0.7 after 5 days	MG-63	Ket loading on Mg-MOF-74	^111^
Mg-MOF-74@MSiO_2_	pore size: 2.0–2.5 nm, thickness ≈ 40 nm	scaffold	ion delivery	not mentioned	not toxic	BMSCs	sol–gel method	^126^
m-Mg-MOF74/n-Mg-MOF74	m-Mg-MOF74:3–4 μm,n-Mg-MOF74:250–350 nm	filler	biocompatibility, osteogenesis, angiogenesis, ion delivery	SD rats	not mentioned	Hela cells, HUVECs or BMSCs	solvothermal synthesis method	^110^
Mg/Zn-MOF74 (AT-Mg/Zn)	particle size of AT-Mg/Zn group: 0.8–7.5 μm, The coating thicknesses of AT-Mg/Zn: 10.7–3.6 μm, WCA of Ti: 62.7 ± 3.2°, WCA of AT: 5.2 ± 1.2°, WCAof AT-Mg/Zn group: 9°	implant	Osteogenesis, antibacterial, anti-inflammatory, ion delivery	Rat femur defect model	not mentioned	RAW264.7	solvothermal synthesis method	^122^
BMSCs@MEGH-D	rod-like, size: 100 nm diameter, ∼700 nm length	Scaffold	osteogenesis, angiogenesis, anti-inflammatory	nude mice and diabetic rabbit skull defect models	>95%	BMSCs	hydrothermal synthesis method	^123^

The detailed comparison of Mg-based MOFs reveals distinct differences in their biological performance, structural attributes, and application potential. Among the MOFs analyzed, the BMSCs@MEGH-D scaffold emerges as the most promising due to its superior cell viability (>95%) and demonstrated efficacy across multiple relevant animal models. This MOF contributes to its excellent osteogenic, angiogenic, and anti-inflammatory properties, rendering it exceptionally appropriate for advanced bone tissue engineering.

Moreover, Mg/Zn-MOF74 (AT-Mg/Zn) provides a well-rounded approach combining osteogenesis, antibacterial activity, ion delivery, and implant use, which was proven in rat femur defect models. Nonetheless, the absence of detailed cell viability data and long-term biological safety profiles poses questions about its comparative biological safety. In contrast, Ket@Mg-MOF-74 offers effective drug delivery with positive cell viability results in MG-63 cells, highlighting its potential as a drug carrier rather than a structural scaffold, as in vivo evaluations are not reported. The Mg-MOF-74@MSiO_2_ and m-Mg-MOF74/n-Mg-MOF74 systems indicate nontoxic behavior and biocompatibility but also lack in vivo data, limiting their current proven applicability.

At the lower end of this spectrum, Mg-MOF-74/MgF_2_ serves mainly as an anticorrosion and hydrophilic coating for Mg alloys, with no evidence of biological testing or cell viability. Its primary function as a surface modifier rather than a biological scaffold categorizes it as the least suitable for direct biomedical applications among the listed materials. These findings suggest that future research should prioritize comprehensive biological assessments alongside physicochemical optimizations to advance the translational potential of Mg-based MOFs, fostering their integration into clinical scaffold design and targeted therapeutic delivery platforms.

#### 4.1.3 The Role of Zr^2+^ and Zr-Based MOFs in Bone Regeneration

Zirconium, a naturally occurring trace element, does not have a clearly identified biological function; however, it is highly valued in biomedical applications due to its notable mechanical strength and excellent biocompatibility.^127,128^ Zr compounds, particularly ZrO, are extensively used in the production of prosthetic devices.^127^ Experimental evidence indicates that Zr^2+^ ions can facilitate bone regeneration by enhancing both the differentiation and proliferation of osteoblasts, especially within a concentration range of 5–50 μM. Additionally, zirconium supports osteoblast mineralization and activates the BMP signaling pathway, which is critical for osteoblast differentiation.^56^ Regardless of these encouraging results, research examining the detailed mechanisms by which zirconium modulates bone cell signaling pathways remains sparse, underscoring the necessity for further investigation to comprehensively elucidate its role in bone regeneration.^129^ Given its capacity to stimulate osteogenesis and promote the expression of osteogenic genes, biomaterials incorporating zirconium show considerable potential to improve the efficacy and longevity of orthopedic and dental implants.^56^

Although clinical trials directly evaluating zirconium supplementation for bone regeneration are limited, zirconium-based materials are frequently studied in clinical contexts involving dental implants, where their influence on osteoblast differentiation is notable. For instance, the combination of zirconia with pyrophosphate-stabilized amorphous calcium phosphate has been demonstrated to substantially enhance the proliferation of osteoblasts at a concentration of 250 mmol/L. Moreover, increased alkaline phosphatase activity reported in these studies reinforces the vital role of zirconium in facilitating osteoblast differentiation and bone formation. These findings collectively underscore the considerable potential of zirconium to enhance the regeneration of bone and promote the clinical success rates of both dental and orthopedic implants.^56,130^

#### 4.1.4 The Role of Ca^2+^ and Ca-Based MOFs in Bone Regeneration

Calcium functions as a critical element within biodegradable calcium phosphate-derived biomaterials specifically designed for applications in the regeneration of bone across trauma surgery, orthopedics, and dentistry.^131–133^ As the mineral that is most prevalent in the human body, calcium fundamentally exists within the skeletal system, serving as the key supplementary element in bone tissue and significantly affecting cellular activities. Extracellular calcium levels in the millimolar range have been demonstrated to enhance the survival, differentiation, and proliferation of osteoblasts and mesenchymal stromal cells derived from bone marrow. The regulation of calcium homeostasis is meticulously controlled by hormones such as calcitonin and parathyroid hormone, which adjust serum calcium levels by either promoting the release of PTH or suppressing calcitonin, thus affecting bone resorption that is mediated by osteoclasts. Throughout the dynamic bone remodeling process, osteoclast-driven resorption can locally increase extracellular calcium ion concentrations to as high as 40 mM^56^. These transient elevations in calcium levels have been discovered to inhibit the resorptive activity of osteoclasts while simultaneously promoting the differentiation and proliferation of osteoblasts and mesenchymal stromal cells, thereby coordinating key mechanisms crucial for regeneration of bone and tissue repair.^75^

Calcium signaling is pivotal in the pathways of bone regeneration, particularly within the Wnt signaling cascade and its downstream effector, the pathway of β-catenin, where calcium functions as a crucial second messenger.^134,135^ Experimental evidence indicates that administration of Wnt11 or Wnt5a significantly increases the frequency of calcium transients in zebrafish embryos, effectively doubling their occurrence.^136^ Furthermore, calcium plays a central role in the interaction between β-catenin-dependent and -independent pathways, mediating the inhibition of β-catenin signaling through both calcium-dependent and -independent mechanisms.^135,137^ Extracellular calcium also activates the CaSR, which is represented in cells derived from hematopoietic and mesenchymal origins.^138^ Elevated calcium concentrations enhance the proliferation, chemotaxis, and osteogenic differentiation of MSCs derived from bone marrow in a dose-responsive way via CaSR activation. This receptor activation triggers phosphorylation of ERK1/2, key elements of the MAPK signaling pathway, thereby regulating cellular proliferation across diverse mammalian cell types.^75,139^ Additionally, CaSR activation initiates PLC signaling,^134^ leading to sustained elevations in cytosolic calcium and subsequent SOCE mediated through the release of calcium from the endoplasmic reticulum mediated by IP3 receptors. Voltage-gated calcium channels further facilitate calcium influx into osteoblasts, promoting the osteogenic differentiation of osteoprogenitor cells.^75,140^

Extracellular calcium concentrations ranging from 3 to 10 mM have been identified as optimal for stimulating bone cell proliferation across multiple species, including humans, pigs, and rats. Concentrations between 10 and 20 mM are similarly effective in promoting osteogenic differentiation.^141^ Clinical studies have highlighted the positive impact of increased calcium intake on the BMD, especially among vulnerable groups. Evidence suggests that elevating calcium intake to approximately 110% of the suggested daily allowance can significantly improve BMD in both adolescent children and postmenopausal women.^56,142^ Importantly, calcium supplementation, frequently combined with vitamin D, has been linked to enhanced bone regeneration and a lower risk of fractures. Recent technological advancements, such as hybrid nanoparticle platforms, have demonstrated the potential for targeted delivery of calcium to osteoporotic bone sites, thereby supporting localized calcium accumulation, bone formation, and osteoblast differentiation. The coadministration of calcium and vitamin D represents a promising therapeutic approach for reducing fracture incidence, highlighting the multifaceted advantages of calcium supplementation in maintaining bone health, particularly within at-risk populations.^56^

Calcium phosphate-based materials have undergone significant development for bone replacement therapies due to their compositional resemblance to natural bone and the essential role of calcium in regulating cellular functions.^131,132,143^ These biomaterials contain various calcium phosphate phases that influence their bioactivity, which is critical for facilitating calcium phosphate binding and leading to localized calcium depletion near the material interface. While the deposition of calcium phosphate on the surfaces of bone substitute materials supports osseointegration, the resulting calcium-deficient microenvironment adjacent to these biomaterials remains not fully understood, particularly taking into account the regulatory effects on osteoblasts and progenitor cells that are dependent on calcium.^144^ Experimental studies have shown that osteoprogenitor cells, including bone-derived MSCs, can adapt and address the deficiency of calcium when cultured in the presence of highly bioactive xerogels; however, the underlying mechanisms remain unclear.^75^ Importantly, the positive influence on cell survival, differentiation, and proliferation observed with highly bioactive composites is primarily attributed to the release of ionic dissolution products such as phosphate ions or silica. Hence, it is hypothesized that optimal osteogenic differentiation and bone formation outcomes are achieved when calcium phosphate-based biomaterials dissociate readily into phosphate and calcium ions, emphasizing the critical interplay between the biomaterial’s bioactivity and cellular responses during bone regeneration.^145^

One of the most important uses of MOFs in the treatment of bone conditions is their application for the delivery of drugs. UiO-66, a zirconium-based MOF, has been utilized for loading various therapeutic agents to address different bone-related diseases. Karakeçili and colleagues fabricated chitosan scaffolds through a wet-spinning process, which were incorporated with UiO-66 nanocrystals loaded with Fosfomycin (CHI/UiO-66/FOS), designed specifically to treat infected bone defects, such as osteomyelitis. These scaffolds possess a three-dimensional fibrous mesh architecture with diameters of fibers ranging from 125 to 155 μm. In vitro studies demonstrated that the CHI/UiO-66/FOS scaffolds effectively eliminate *Staphylococcus aureus* bacteria. Furthermore, these scaffolds exhibited biocompatibility with MC3T3-E1 preosteoblast cells, facilitating the upregulation of genes associated with bone formation and enhancing extracellular matrix mineralization. Collectively, these results indicate that UiO-66 can perform multifunctional roles. The promising therapeutic potential of CHI/UiO-66/FOS scaffolds as a novel intervention for infected bone defects such as osteomyelitis warrants further research.^146^

As previously noted regarding the vital role of calcium, the composite C_2_S@PCN-224 developed by Du et al. exemplifies the potential benefits of combining Zr-based MOFs with calcium for patient treatment. In this study, PCN-224, a subclass of MOFs, was synthesized and deposited onto the surfaces of 3D-printed porous β-Ca_2_SiO_4_ (C_2_S) scaffolds by using a hydrothermal synthesis technique. SEM images revealed the scaffolds’ surfaces featured uniform, interconnected pores approximately 400 μm in diameter. Furthermore, the composite scaffolds (C_2_S@MOFs) exhibited a porosity of around 90%, which is higher than the 71.9 ± 0.3% porosity of the pure C_2_S scaffolds, attributed to the formation of nanoscale particles. Zeta potential analysis indicated an enhancement in the surface potential of the composite scaffolds corresponding to higher reactant concentrations. These enhancements collectively suggest improved chemical stability and structural properties conducive to bone regeneration applications.

In this study, the osteogenic potential and biocompatibility of the scaffolds were evaluated by examining the proliferation, adhesion, and osteogenic gene expression of rBMSCs. Live/dead staining revealed that the cells exhibited stretched morphologies with visible filamentous and lamellar pseudopods, indicating that the modification of PCN-224 improved the biocompatibility of the C_2_S scaffolds. Furthermore, the chemical stability and high porosity conferred by the PCN-224 coating contributed to the improved biocompatibility of the composite scaffolds. ALP activity, a marker of osteogenic differentiation, was notably elevated in cells cultured on the composite scaffolds. In addition, the expression levels of key osteogenic genes, including OCN, OPN, ALP, and RUNX2, were upregulated. Moreover, in vivo studies demonstrated that the composite scaffolds facilitated enhanced healing of rat cranial bone defects compared to C_2_S scaffolds alone.^147^

Another Zr-based MOF nanocomposite, Fe_3_O_4_@CS@UIO-66-NH_2_(Zr), was synthesized through a multistep process beginning with the preparation of magnetite nanoparticles (Fe_3_O_4_), followed by forming the Fe_3_O_4_@CS core–shell by sonicating magnetite particles with chitosan in a water–ethanol mixture and stirring at 35 °C. The final nanocomposite was obtained by functionalizing Fe_3_O_4_@CS with succinic anhydride through sonication and reflux in water to introduce carboxylic groups, then mixing with zirconium chloride and 2-aminoterephthalic acid in acetic acid and dimethylformamide, followed by sonication and reflux at 110 °C, with the product washed and dried under vacuum.

The synthesized Zr-MOF nanocomposite was rigorously characterized, confirming its successful integration and functionality. FT-IR spectroscopy verified the presence of characteristic Zr–O bonds and framework vibrations, while XRD patterns confirmed the crystalline structure of UIO-66-NH_2_ grown on the magnetic core. FE-SEM and EDX mapping illustrated a nonuniform but interconnected morphology with homogeneous distribution of all elements, including zirconium. BET verified the presence of a mesoporous structure characterized by a significant surface area (91.182 m^2^/g), which significantly decreased after drug loading, indicating pore occupancy. TGA showed an enhanced weight loss in the drug-loaded sample, corroborating successful encapsulation. The nanocomposite exhibited superparamagnetic behavior with reduced saturation magnetization post-MOF growth, yet was sufficient for magnetic targeting.

The nanocomposite exhibited pH-sensitive drug loading and release behavior for pantoprazole, attaining a drug loading efficiency of 79% in acetate buffer (pH 5.0) and 75% in deionized water over a period of 48 h, with corresponding drug loading contents of approximately 14% and 10%. Adsorption isotherms followed the Langmuir and Freundlich models, indicating multilayer adsorption on active sites, while release kinetics adhered to the pseudo-second-order model. Owing to its magnetic characteristics enabling external field-guided targeting, porosity facilitating drug encapsulation, and biocompatibility from chitosan coating, the Fe_3_O_4_@CS@UIO-66-NH_2_(Zr) demonstrated potential as a nanocarrier for targeted delivery, minimizing side effects by reducing required dosages.^148^ Table 3 summarizes recent applications of Zr-based MOFs in the treatment of bone diseases.

**Table 3:** Zr-Based MOFs Studied in Bone Disease Treatment

MOF diversity	characteristics of MOFs	type of function	application	In vivo test	cell viability	cell name	method of synthesis	refs
C_2_S@ PCN-224	Zeta potential of C_2_S@MOF: 27.9–30.8 mV, Zeta potential of C_2_S:15.6 mV, size: macropores (≈400 μm), the porosity of C_2_S scaffold: 71.9 ± 0.3%, The porosity of C_2_S@MOF:88–91%	scaffold	bioactivity, osteogenesis	male (SD) rats	not mentioned	RBMSCs	hydrothermal synthesis of MOF on 3D-printed C_2_S	^147^
F-doped MOF-801/Ti	coating thickness: Zr-MOF: 5.38–14.13 μm	implant	bioactivity, biocompatibility, osteogenesis, antibacterial	rat cranial bone defect	not mentioned	MSCs	solvothermal synthesis method	^149^
CHI/UiO-66/FOS	average fiber diameter: 125–155 μm	scaffold	biocompatibility, osteogenesis, antibacterial	not mentioned	not mentioned	MC3T3-E1 preosteoblasts	wet spinning synthesis method	^146^
UiO-66	particle size: 170 nm	scaffold	Osteogenesis	rabbit femoral condyle defect model	not toxic >100%	HFOB cells	solvothermal synthesis method	^150^
Fe3O4@CS@UIO-66-NH2(Zr)	surface area: 91.182 m^2^/g	nanocarrier	ion delivery	not mentioned	not mentioned	not mentioned	step-by-step preparation of MOF	^148^

Based on the specific table data, C_2_S@PCN-224 emerges as the best performer in terms of porosity and scaffold structure, with its high porosity (88–91%) supporting excellent osteogenic potential in vivo. F-doped MOF-801/Ti ranks highly for its effective biocompatibility, antibacterial properties, and osteogenesis, making it a strong candidate for implant coatings. UiO-66 is notable for its nanoscale particle size and excellent cell viability (>100%) in rabbit bone defect models, confirming its safety and osteogenic function. CHI/UiO-66/FOS also shows good biocompatibility and antibacterial function but lacks complete in vivo data, while Fe_3_O_4_@CS@UIO-66-NH_2_, focusing on ion delivery, is the least validated due to limited biological and in vivo evidence.

Looking forward, these Zr-MOFs demonstrate multifunctionality essential for bone disease treatment, such as the combination of osteogenesis, antibacterial action, and biocompatibility. Future research should focus on enhancing multifunctionality, optimizing scaffold porosity, and expanding robust in vivo and clinical validations. Their tunable structure and synthesis methods offer promising opportunities to create tailored biomaterials that promote bone repair, combat infections, and potentially deliver therapeutic agents simultaneously. Zr-MOFs, with these advances, are poised to become highly effective platforms in regenerative medicine and bone disease therapy.

#### 4.1.5 The Role of Fe^2+^ and Fe-Based MOFs in Bone Regeneration

Iron is a vital ion within the human body, playing a critical role in various cellular processes such as the synthesis of proteins, RNA, and DNA, as well as facilitating cellular proliferation, differentiation, and electron transport.^151,152^ It functions as an essential part of many enzymes, including oxidases, peroxidases, aconitases, catalases, ribonucleotide reductases, and nitric oxide synthases.^153,154^ Unlike zinc and magnesium, iron acts as a growth-limiting factor, because its propensity to create insoluble oxides when oxygen is present restricts the absorption of Fe^3+^ ions. To effectively utilize iron for synthesizing oxygen-transport proteins, the body converts insoluble Fe^3+^ to its soluble Fe^2+^ form. This conversion generates free radicals, which can damage nucleic acids, carbohydrates, proteins, and lipids, leading to altered intracellular signaling pathways and potential cell death.^56,155^

Iron is a vital trace element necessary for the transport of oxygen and the regulation of various metabolic enzymes in the human body. It acts as a coordinating ion within myoglobin and hemoglobin and is critical for the hydroxylation of lysine and proline residues in collagen precursors through the enzymes procollagen lysine hydroxylase and procollagen proline hydroxylase.^156^ Inadequate levels of iron may result in anemia and decreased BMD. Conversely, excess iron can impair osteoblast function and extracellular matrix mineralization due to the generation of reactive oxygen species. These ROS promote RANKL activation, which affects Wnt signaling pathways and thereby influences both bone formation and resorption.^157^ Elevated iron (Fe^2+^) or ROS derived from iron motivates the proliferation and differentiation of osteoclast precursors and enhances mature osteoclast activity via M-CSF and RANKL through NFATc1 expression. Conversely, the overabundance of iron and ROS has a detrimental effect on mesenchymal stem cell osteogenic capacity and mature osteoblast function by inhibiting BMP and Wnt signaling pathways, essential for the transcription of Runx2 and Osx. As a result, iron is not regarded as an optimal agent for the treatment of osteoporosis because of its dual impact on bone metabolism.^56,157,158^

In vitro studies have demonstrated that excessive iron accumulation inhibits osteogenic differentiation in human osteoblasts and reduces mineralization. This inhibitory influence is mainly ascribed to the generation of ROS, which impairs both osteoblast function and extracellular matrix mineralization, a phenomenon also observed in vivo in zebrafish larvae.^157^ The negative consequences of surplus iron on osteoblastic markers and bone mineralization can be alleviated by iron chelators such as deferoxamine, which systemically reduce iron levels.^159^ In a similar manner, hepcidin, which regulates iron uptake, alleviates the adverse effects of excess iron on bone formation; however, downregulation of hepcidin leads to elevated iron levels, thereby exacerbating osteogenic dysfunction. These findings underscore the delicate balance of iron homeostasis required to maintain healthy bone formation and highlight potential therapeutic avenues for mitigating iron-induced bone impairment.^75,160^

Exposure of HBMSCs to iron at a concentration of 50 μM inhibits their differentiation along the osteogenic lineage and diminishes the mineralization of the extracellular matrix, as confirmed by in vivo studies in mice. This inhibitory effect appears to be specific to osteogenesis, with no significant impact on chondrogenesis or adipogenesis.^158,161^ Additionally, iron has been observed to promote osteoclast formation, underscoring its potentially detrimental role in biomedical tissue engineering. In contrast, iron oxide nanoparticles have shown a capacity to enhance osteogenic differentiation of human BMSCs in vitro through the activation of the MAPK signaling pathway, suggesting that nanoparticle formulations may help mitigate the negative effects of iron.^162^ Furthermore, a study by Zhao et al. revealed that while increasing iron concentrations suppresses osteoblast activity in a dose-dependent manner, mild iron deficiency can enhance cellular function, whereas severe iron deficiency completely inhibits osteoblastic differentiation.^163^ Elevated iron levels also stimulate osteoclastogenesis and inhibit osteogenic stimuli, presenting challenges for the use of iron in tissue engineering applications.^164^ These findings highlight the need for further research to clarify the potential therapeutic benefits and risks of iron in this field.

Osteoarthritis is a chronic degenerative disorder that necessitates effective drug delivery systems for treatment. Xiong et al. developed a pH-responsive drug delivery carrier named MOF@HA@PCA, specifically designed for OA therapy. This system utilizes MIL-100­(Fe), characterized by its dense pores and large pore sizes, making it an excellent candidate for drug delivery. However, due to its inherent poor hydrophilicity, MIL-100­(Fe) was modified with the hydrophilic polymer HA. Subsequently, the anti-inflammatory agent PCA was loaded onto the preprepared MOF@HA nanoparticles through 24 h of shaking in the dark.

TEM revealed that the size of these MOF nanoparticles was approximately 100 nm [Figure 7(a1–a3)], and DLS measurements in deionized water showed a hydrodynamic size of 123.4 nm for the MOF@HA@PCA nanoparticles [Figure 7a_7_)]. The successful and uniform modification of MOF nanoparticles by HA was confirmed through spectroscopic analyses, including FTIR, XRD, TEM, and DLS [Figure 7a_1_–a_5_, a_7_)]. Furthermore, the zeta potential of the nanoparticles decreased from −9.3 mV for the bare MOFs to −12.1 mV after HA modification and further to −21 mV upon PCA loading, indicating enhanced water stability beneficial for drug delivery applications [Figure 7(a_8_)].

**Figure 7:**
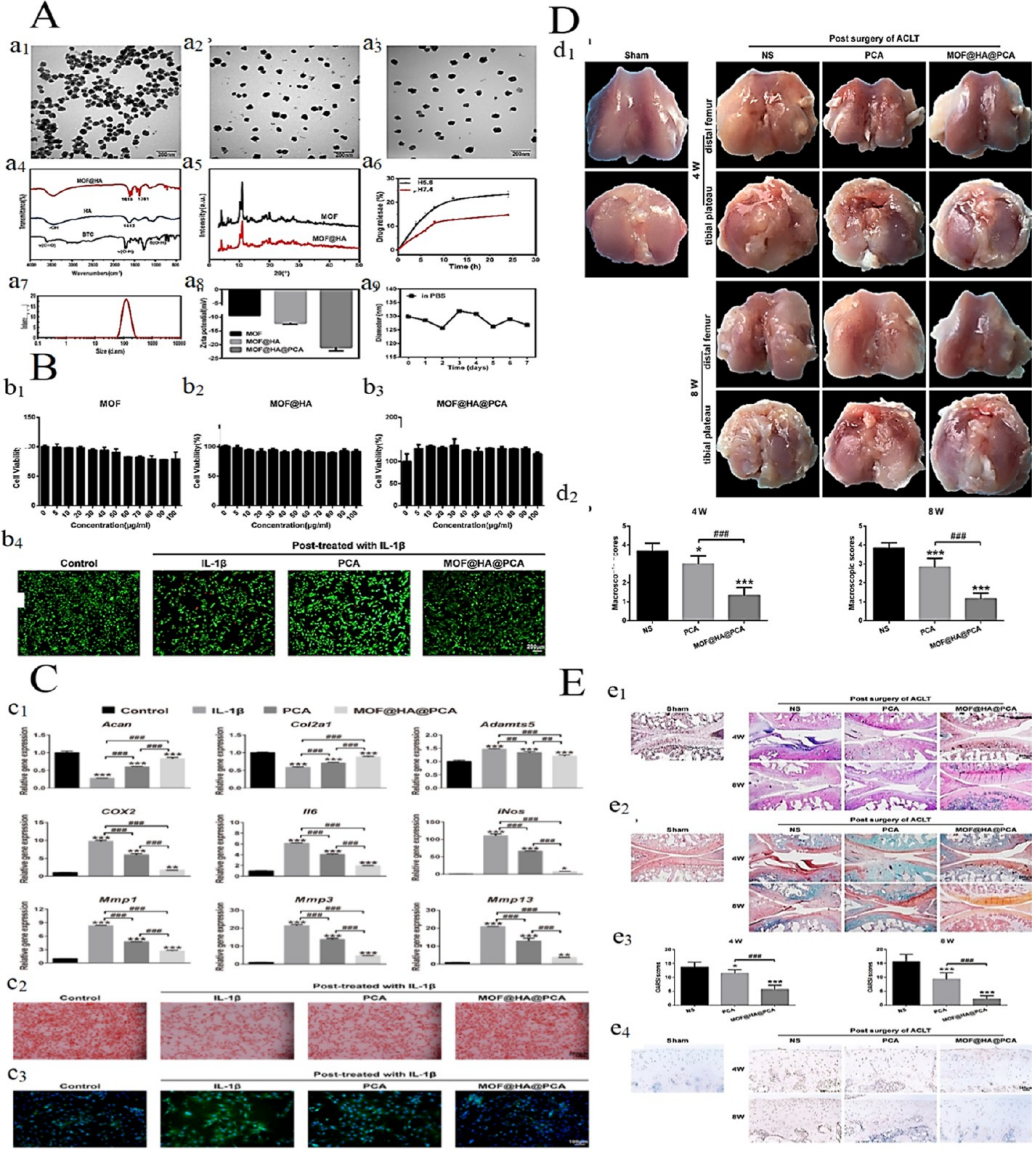
Fabrication of MOF@HA@PCA for drug delivery. (A) (a_1_) TEM images of MOF, (a_2_) MOF@HA, and (a_3_) MOF@HA@PCA. (a_4_) FT-IR spectra. (a_5_) XRD spectra. (a_6_) Cumulative in vitro drug release profile. (a_7_) DLS. (a_8_) Zeta potentials. (a_9_) The stability analysis of MOF. (B) (b_1_–b_3_) Cytotoxicity of MOF, MOF@HA, and MOF@HA@PCA in chondrocytes. (b_4_) Cell viability. (C) (c_1_) In relation to mRNA levels of chondrogenic markers (Col2a1, Acan) and OA-relative genes (Mmp13, Mmp3, Mmp1, iNos, Adamts5, COX2, and Il6). (c_2_) Safranin O stained for GAG production. (c_3_) The expression of MmP-13 was identified through immunofluorescent staining. (D) (d_1_) Macroscopic appearance. (d_2_) Macroscopic scores of the distal femur and tibial plateau from rats. (E) (e_1_) Hematoxylin and eosin (HE). (e_2_) Safranin O/fast green staining of cartilage. (e_3_) OARSI scores for histology of articular cartilage. (e_4_) Immunohistochemical staining of MmP-13 in cartilage.^165^ Reproduced from ref 165. Available under a CC-BY 4.0 license. Copyright 2020 Xiong et al.

The ratio of drug loading and the efficiency of encapsulation of the MOF@HA@PCA nanoparticles were measured at 19.4% and 38.8%, respectively. Investigation of their drug release behavior revealed a controlled and sustained release of PCA, which was pH-dependent; at pH 5.6, degradation of the MOF structure significantly enhanced the anti-inflammatory efficacy of PCA [Figure 7(a_6_)]. Cellular proliferation assays using MTT demonstrated that PCA-loaded nanoparticles exhibited low cytotoxicity, with maximal chondrocyte viability observed at a concentration of 30 μg/mL (Figure 7B). Moreover, the MOF@HA@PCA nanoparticles effectively down-regulated inflammatory markers while upregulating cartilage-specific markers in vitro (Figure 7C). In vivo studies utilizing a rat OA model showed that the IA injection of these nanoparticles markedly reduced inflammatory cytokines and promoted cartilage repair. These findings suggest that MOF@HA@PCA nanoparticles represent a promising therapeutic approach for OA management following IA (Figure 7D,E).^165^ Table 4 provides a summary of recent applications of iron-based nanomaterials in bone disease treatment.

**Table 4:** Fe-Based MOFs Studied in Bone Disease Treatment

MOF diversity	characteristics of MOFs	type of function	application	in vivo test	cell viability	cell name	method of synthesis	refs
Alen@β-CD@Fe-MIL-88B@Hap	pore size of Fe-MIL −88B: 5 nm, size of Fe-MIL −88B: 50 and 200 nm, surface area of Fe-MIL −88B: 187.91 m^2^/g, surface area of Alen@β-CD@Fe-MIL-88B: 74.30 m^2^/g	carrier	drug delivery	not mentioned	not mentioned	not mentioned	Alendronate-loaded Fe–MOF encapsulated in porous hydroxyapatite	^166^
MBG/MOF	size of MBG/MOF: 400 μm	scaffold	antibacterial, biocompatible, bioactive, treating osteoarticular tuberculosis	not mentioned	not mentioned	HBMSCs	3D-printing synthesis method	^167^
MOF@HA@PCA	size: 123.4 nm, Zeta potential of MOF@HA@PCA: −21 mV	carrier	anti-inflammatory, drug delivery, biodegradable	distal femur and tibial plateau of SD rats	not toxic	chondrocytes	PCA loading on presynthesized MOF@HA	^165^
HA@MOF/d-Arg	size of HA@MOF/d-Arg: 117 nm	nanoparticle	antitumor, ROS producer, biocompatible	BALB/c mice	toxic to the cancer cell line	F K7M2 osteoblast cell line	d-arginine-loaded MOFs encapsulated in hyaluronic acid	^168^
Mg@NH2-MIL-100(Fe)-PAA	not mentioned	not mentioned	ion delivery, osteogenesis	not mentioned	not mentioned	not mentioned	Mixing activated PAA, MgCl_2_ and presynthesized NH2-MIL-100(Fe)	^169^

Based on the table about Fe-MOF in bone disease treatment, the best-performing Fe-based MOFs vary by functional parameters. The MBG/MOF scaffold demonstrates the best overall potential due to its large size (400 μm), antibacterial, bioactive, and biocompatible properties, along with its specific application in treating osteoarticular tuberculosis and support for HBMSCs. This makes it highly promising for infection-associated bone repair. MOF@HA@PCA nanoparticles also show excellent prospects as they combine anti-inflammatory, drug delivery, and biodegradable functions with proven in vivo biocompatibility in rat models, making them suitable for inflammatory bone and cartilage diseases. HA@MOF/d-Arg shows strong potential for bone tumor treatment due to its antitumor activity and selective toxicity to cancer cells, combined with osteoblast compatibility, though its application may be more specialized.

Conversely, the Alen@β-CD@Fe-MIL-88B@Hap drug carrier, despite good physicochemical properties (pore size and surface area), has no reported in vivo or cell viability data, limiting its immediate translational potential. Mg@NH2-MIL-100­(Fe)-PAA, focusing on ion delivery and osteogenesis, remains the least validated due to a lack of biological and in vivo evidence.

Future research must fill gaps in in vivo validation and explore combinations that integrate ion delivery, inflammation modulation, and tumor suppression to maximize the therapeutic impact. Overall, Fe-based MOFs with robust multifunctionality and validated biocompatibility present the best prospects for advancing bone disease treatments.

#### 4.1.6 The Role of Cu^2+^ and Cu-Based MOFs in Bone Regeneration

Copper is a crucial trace element in human physiology, existing predominantly in the ionic forms Cu^+^ and Cu^2+^. It is essential in the process of synthesis of copper-containing proteins that serve enzymatic functions needed for electron transfer reactions, oxygen transport, and metal ion storage.^170,171^ Furthermore, copper is indispensable for metabolism and remodeling of bone, where it promotes the deposition of collagen fibers, supports the production of new blood vessels, and facilitates the osteogenic differentiation of mesenchymal stem cells­(MSCs).^56,172^ Its recognized antibacterial properties also contribute to its significance in bone regeneration. Clinical studies have demonstrated that copper supplementation, for instance, at a dosage of 3 mg/day, can attenuate BMD loss in middle-aged women over extended periods. Additionally, combined supplementation with calcium, zinc, manganese, and copper has been shown to preserve spinal bone density in elderly women, whereas placebo recipients exhibited declines in bone density. However, the evidence remains mixed, as other research has reported no substantial benefit on whole-body bone content with a lower copper supplementation (2 mg) alongside calcium and zinc in postmenopausal women.^56^

Research based on scaffold studies indicates that copper influences bone metabolism by activating the β-Catenin signaling pathway, which enhances the expression of osteogenesis-related genes. Although few studies directly examine the role of copper in bone regeneration, animal experiments have indicated that copper may downregulate Wnt signaling pathways.^173–175^ Dysregulation of copper levels is also known to impact the nervous system and cause vascular abnormalities. Extensive studies on copper deficiency have demonstrated its fundamental importance for skeletal growth and development. The growing interest in the function of copper in bone regeneration can be attributed to its antibacterial characteristics and its ability to enhance collagen fiber deposition and angiogenesis, both of which are essential for the formation of vascularized tissue.^75,176^ Additionally, studies on copper-doped silicate bioceramics report positive effects on angiogenic growth factors within human cells, suggesting that the release of Cu^2+^ from bioactive glass may promote bone ingrowth into scaffold structures.^56^

Current evidence supports copper’s influence on enhancing the osteogenic differentiation of MSCs. Initial studies report that copper reduces MSC proliferation but simultaneously doubles their differentiation into osteoblasts and increases calcium deposition, despite a decrease in alkaline phosphatase activity.^177^ Similar findings in rat MSCs reveal that copper suppresses osteogenic differentiation and alkaline phosphatase activity, leading to reduced bone nodule formation and cytoskeletal abnormalities. In rat models, copper impaired ectopic bone formation while promoting vascularization in regenerated soft tissue, though collagen formation was inhibited.^172^

A study examining preosteoblastic MC3T3-E1 cells cultured on copper-containing bioglasses revealed a concentration-dependent effect of copper on cellular behavior. Scaffolds doped with CuO at concentrations ranging from 0.4 to 0.8 wt % exerted no significant influence on cell proliferation or alkaline phosphatase activity. However, increasing CuO concentration to 2.0 wt % resulted in a marked reduction in both parameters. In vivo experiments involving rat calvarial defects demonstrated that elevated concentrations of Cu^2+^ ions significantly impeded new bone formation, reducing bone regeneration from 46 ± 8% to 0.8 ± 0.7%. Conversely, lower copper concentrations did not show any adverse effects.^178^ Notably, copper positively affected neovascularization, with the greatest angiogenic response observed at 2.0% CuO. Additional research reported that chitosan scaffolds doped with copper considerably enhanced bone volume in critical-sized calvarial defects in rats, as evidenced by micro-CT analysis, effectively doubling bone volume compared to copper-free scaffolds.^27,75,179^

Due to the high risk of bone tumor recurrence following surgical resection, considerable attention has been directed toward developing multifunctional materials that can simultaneously eradicate residual tumor cells and facilitate bone defect repair.^180^ Dang and colleagues fabricated Cu-TCPP-TCP nanosheets by integrating 3D printing with an in situ solvothermal synthesis method to address these dual therapeutic goals. Characterization via optical microscopy revealed that the scaffold color deepened to a darker orange-red with increasing Cu-TCPP concentrations in the reaction solutions. SEM analysis showed the nanosheet coating thickness to be approximately 50 nm.

The most effective modified form of the synthesized Cu-TCPP-TCP scaffold demonstrated potent cytotoxicity against LM8 tumor cells by inducing hyperthermia during exposure to NIR light. Importantly, the scaffold exhibited no cytotoxic effects in the absence of light irradiation. In vitro studies further revealed that these scaffolds significantly enhanced the expression of osteogenic and angiogenic genes, thereby promoting differentiation of HBMSCs and HUVECs. Evaluation using critical-sized femoral defect models in rabbits via micro-CT scans showed that although the hyperthermia generated by the scaffold caused localized damage to some normal bone tissue, unaffected bone cells migrated into the scaffold, proliferated, and differentiated into new bone marrow. These findings underscore the scaffold’s dual capacity to stimulate osteogenesis and angiogenesis while effectively eradicating bone tumor cells. Consequently, the study demonstrates that Cu-TCPP-TCP scaffolds implanted in bone defects can efficiently ablate bone tumors and inhibit their growth under NIR light irradiation.^181^

While developing nanozymes to scavenge ROS holds potential as an innovative therapeutic approach for OA, significant challenges remain. Chief among these is the inherently limited antioxidant capacity exhibited by many nanozymes. Zhou et al. synthesized a copper-based metal–organic framework (Cu-MOF) nanozyme via a self-assembly technique (Figure 8). This method enabled the formation of uniform CuNx active sites through coordination between copper and nitrogen atoms from the 4,4′-bipyridine linkers.

**Figure 8:**
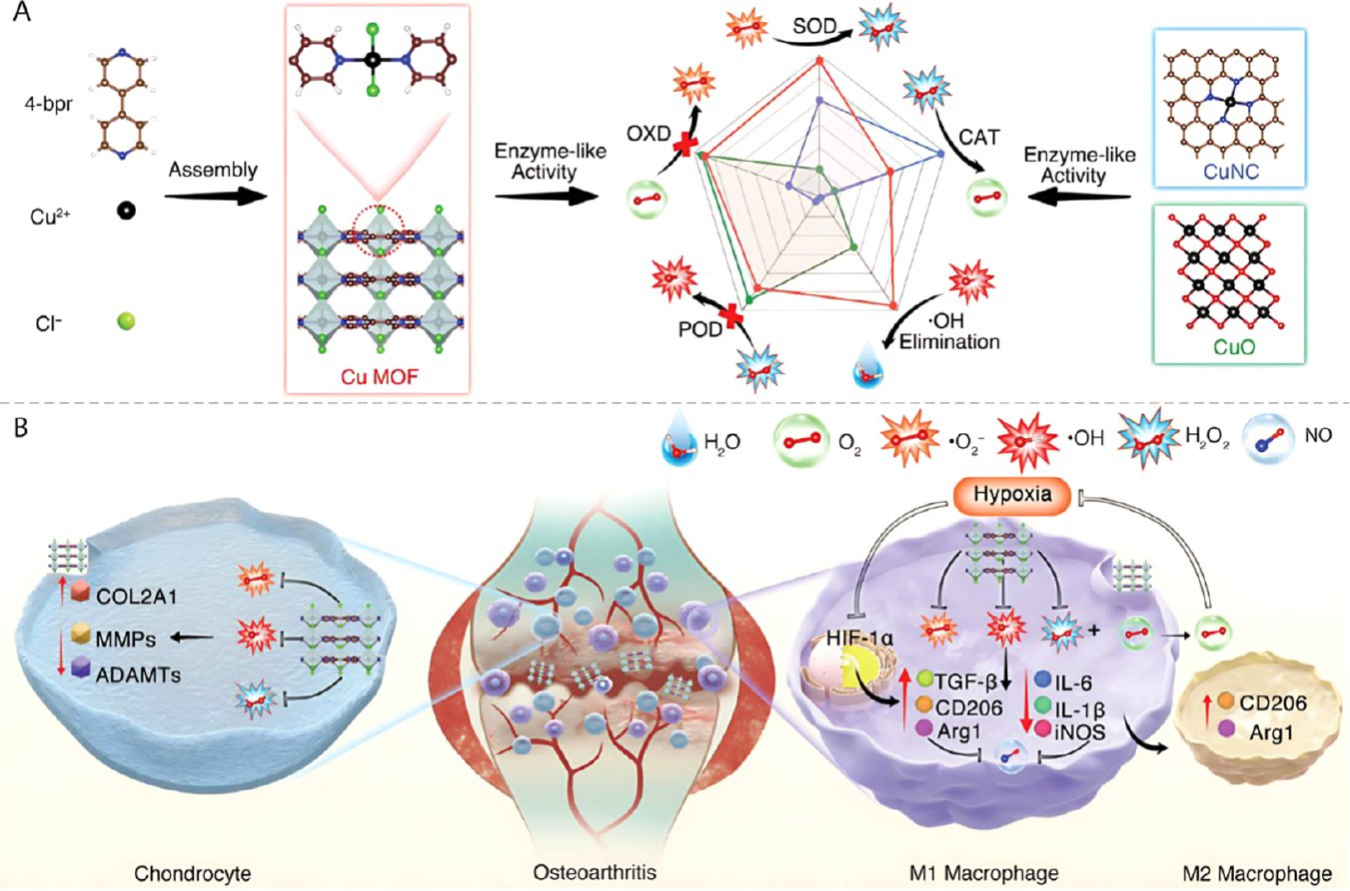
Cu MOF as a comprehensive and potent antioxidant nanozyme for efficient OA treatment. (A) Schematic illustration of the synthesis of Cu MOF nanozyme, highlighting its enzyme-like catalytic activities compared with those of other copper-based nanozymes (Cu nanoclusters and CuO). (B) Diagram depicting the therapeutic mechanism of Cu MOF nanozyme in OA treatment through the promotion of macrophage polarization to the anti-inflammatory M2 phenotype by reducing intracellular ROS and improving hypoxia, thereby inhibiting synovitis and cartilage degeneration.^182^ Reproduced from ref 182. Available under a CC-BY 4.0 license. Copyright 2024 Yu et al.

The resulting nanozyme displayed cubic morphologies approximately 1.5 μm in dimension, as revealed by TEM and SEM. Key characterization findings include a redshift of the UV–visible absorption peak for 4,4′-bipyridine to 388.48 nm, indicative of CuNx site formation; XRD patterns consistent with predicted crystalline structures; and EDS confirming homogeneous copper and nitrogen distribution.

In terms of biological behavior, the Cu MOF nanozyme demonstrated broad-spectrum antioxidant capabilities, including efficient scavenging of hydroxyl radicals while exhibiting minimal pro-oxidant effects. This was supported by DFT calculations, which revealed low energy barriers for disproportionation and ROS. In vitro experiments showed that the nanozyme effectively reduced intracellular ROS levels and alleviated hypoxia in synovial macrophages, facilitating their polarization from the pro-inflammatory M1 phenotype to the anti-inflammatory M2 phenotype. This phenotypic shift contributed to diminished secretion of pro-inflammatory cytokines and protection against cartilage degradation.

In vivo studies using osteoarthritis models further validated the nanozyme’s therapeutic efficacy, demonstrating reduced synovial inflammation and extracellular matrix deterioration, attributed to combined ROS clearance and improved oxygen delivery. Importantly, the nanozyme exhibited excellent biocompatibility without inducing adverse pro-oxidant side effects.^182^ The recent applications of copper-based MOFs in bone disease treatments are summarized in Table 5.

**Table 5:** Cu-Based MOFs Studied in Bone Disease Treatment

MOF diversity	characteristics of MOFs	type of function	application	in vivo test	cell viability	cell name	method of synthesis	refs
Mg-PCL-MOF (folic-acid modified HKUST-1)	size: 1–1.5 μm	implant	biocompatible, ion delivery, osteogenesis	not mentioned	94.6%	MC3CT-E1	conventional solution method	^183^
NO-loaded HKUST-1/PCL/Gelatin	size: 53.96 ± 12.53 nm, surface area:1194.77 m^2^/g, pore size: 2.122 nm	scaffold	angiogenesis, tendon regeneration, ion delivery, no delivery, biocompatible	SD rats with a patellar tendon defect	Abs = 2.9 after 7 days (not toxic)	HUVECs	coaxial electrospinning synthesis method	^184^
HKUST-1/folic acid NPs into pectin/PEO	size: 217.3 ± 34.4 nm, Zeta potentials: Hkust-1:11.9, F-Hkust: 13.6 mV	scaffold	ion delivery, folic acid delivery, biocompatible, antibacterial	not mentioned	>80%	L929 mouse fibroblast cells	electrospinning synthesis method	^185^
Cu-TCPP-TCP	Coating thickness: about 50 nm	scaffold	photothermal effect, ion delivery, folic acid delivery, antiosteosarcoma, angiogenesis, osteogenesis	Osteosarcoma mouse model, rabbit femoral defect model	>90%	HBMSCs and HUVECs	3D printing technique with the in situ growth method in a solvothermal system	^181^
Cu MOF	Cubic, size: roughly 1.5 μm	nanozymes	antiosteoarthritis, anti-inflammatory	collagenase-induced osteoarthritis model	no mentioned	Raw264.7, chondrocyte	self-assembly method	^182^

According to the table, the best performer in terms of biocompatibility, osteogenesis, and ion delivery appears to be the Mg-PCL-MOF (folic acid-modified HKUST-1), which demonstrates high cell viability (94.6%) with MC3T3-E1 osteoblast cells, although detailed in vivo studies were not mentioned. The NO-loaded HKUST-1/PCL/Gelatin scaffold excels at promoting angiogenesis and tendon regeneration in rat models, showing effective nitric oxide (NO) delivery and excellent biocompatibility with human endothelial cells, highlighting its potential for vascularized tissue repair. The Cu-TCPP-TCP scaffold stands out for its multifunctionality, including photothermal effect, ion and folic acid delivery, antiosteosarcoma activity, angiogenesis, and osteogenesis, with robust in vivo results and cell viability above 90%, making it highly promising for both tumor treatment and bone regeneration.

In contrast, HKUST-1/folic acid nanoparticles offer reasonable biocompatibility and antibacterial action but have lower cell viability (>80%) and lack in vivo data, placing them as moderately effective. The Cu MOF nanozymes for antiosteoarthritis treatment show promise as anti-inflammatory agents in osteoarthritis models but have limited detailed data about cell viability or bone regeneration directly.

From a prospect perspective, Cu-based MOFs hold considerable promise driven by their strong antibacterial, angiogenic, osteogenic, and antitumor functionalities, which are critical for multifaceted bone disease therapies. Their high surface area and ion release dynamics support drug loading and sustained therapeutic action. However, stability issues in aqueous environments and potential cytotoxicity at high copper concentrations necessitate further optimization. Future research should focus on balancing biological efficacy and safety, improving in vivo validations, and expanding multifunctional designs combining osteogenesis, inflammation modulation, and anticancer effects to maximize clinical translation.

#### 4.1.7 The Role of Co^2+^ and Co-Based MOFs in Bone Regeneration

Cobalt is an essential trace element involved in various physiological processes, notably as a component of cobalamin (vitamin B12), which stimulates red blood cell production and promotes angiogenesis by activating HIF.^186^ Medical CoCr alloys, commonly employed in metal-on-metal hip prostheses, have exhibited improved wear resistance when modified by nitrogen PIII. However, this modification also increases the in vitro release of Co (II) ions. Studies investigating the impact of Co (II) ions on MSC osteogenic differentiation indicate that these ions influence the process. Specifically, the modified CoCr alloy upregulates osteopontin expression through a hypoxic response in both naive and differentiated MSCs, though osteocalcin production varies between pristine and modified alloys.^187^ While earlier reports suggested that Co^2+^ ions from CoCr surfaces negatively affect osteogenic lineage differentiation, recent evidence implicates Cr^3+^ ions as the primary contributors to such adverse effects.

Cobalt has been integrated into biomaterials such as calcium phosphate coatings, nanoparticles, hydrogels, and bioglass scaffolds, which locally release Co^2+^ ions at concentrations ranging from 2 to 5 mg/L/day.^56,188^ Mechanistically, cobalt activates Wnt/β-catenin signaling and suppresses Notch signaling, thereby facilitating bone regeneration. Animal studies with cobalt-doped bioactive glass scaffolds demonstrate enhanced angiogenesis and bone formation potential, indicating promise for implant applications.^56,189^ Co^2+^ ions incorporated into calcium phosphate coatings on PLA particles have similarly increased vascularization without inducing pathological inflammation in goat models. Although Co^2+^ ions have been reported to impair cell viability and disrupt cytoskeletal organization in MC3T3-E1 osteoblastic cells, cobalt-doped hydroxyapatite nanoparticles have accelerated osteogenesis and bone repair in osteoporotic models.^75,190^ Collectively, these findings underscore cobalt’s potential as a multifunctional agent in bone regenerative therapies, yet they also highlight the necessity for further research to elucidate its safety profile and optimize its clinical applicability.

Studies have demonstrated that Co^2+^ incorporated into HAp nanoparticles, when combined with blood or PRGF, significantly enhances osteoblast proliferation, mineralization, and bone regeneration. This synergistic effect is believed to arise from the capacity of blood and PRGF to compensate for impaired growth factor expression and osteogenic differentiation in hMSCs exposed to Co2+.^191,192^ These findings suggest that bone mineral-containing scaffolds are highly compatible with cobalt doping, as cobalt supports rather than inhibits mineralization processes. A similar synergistic enhancement of bone repair was observed in hydrogels doped with Co^2+^ and BMP2, which notably increased bone volume, surface area, and density in rat models.^193^ Furthermore, cobalt-containing bioglasses demonstrated improved collagen deposition, new bone formation, and bone hardness in critically sized defects in rabbits. This regenerative effect was further amplified by the addition of strontium, underscoring the potential of multielement-doped biomaterials in bone tissue engineering.^194^

The one-pot synthesis method involves the incorporation of therapeutic molecules concurrently with the formation of MOFs, ensuring the homogeneous distribution of the agents within the MOF structure and preventing rapid drug release. This method is especially advantageous for fabricating nanomedicines targeting bone regeneration and bone disease treatments. For instance, risedronate has been successfully encapsulated in ZIF-8 MOFs with an encapsulation efficiency of approximately 64%.^39^ Similarly, 4-chloroN-cyclohexyl-*N*-(phenylmethyl)-benzamide was loaded into the cobalt-based MOF, ZIF-67, demonstrating a sustained and favorable drug release profile. These studies underscore the potential of cobalt ions and innovative drug-loading strategies, such as one-pot synthesis, to advance the development of effective MOF-based therapeutics for bone regeneration.^39,195^

Co-TCPP/CPC, a multifunctional injectable calcium phosphate cement (CPC) modified with cobalt-coordinated tetrakis­(4-carboxyphenyl) porphyrin (Co-TCPP), has been developed for the treatment of neoplastic bone defects resulting from bone tumor resection. This composite biomaterial was synthesized via a bottom-up solvothermal method, incorporating Co-TCPP nanoparticles into cement powders. TEM and AFM analyses revealed that the 2D Co-TCPP nanoparticles exhibit an average lateral size of approximately 400 nm and a thickness ranging between 10 and 20 nm. Besides, the SEM images of Co-TCPP/CPC nanoparticles illustrated that the addition of Co-TCPP, regardless of the content, did not have a serious effect on the phase composition and morphology of CPC.

The release behavior of Co^2+^ ions from Co-TCPP/CPC and the degradation of the MOF structures were studied by immersion in Tris–HCl buffer (pH 7.4). Although Co-TCPP MOFs are coordinated through bonds between Co^2+^ ions and TCPP ligands, these coordination bonds exhibit relatively weak stability in this buffer, leading to progressive degradation and increased cobalt ion release over time (Figure 9A). Under 808 nm NIR laser irradiation, the Co-TCPP/CPC demonstrated effective photothermal performance, unlike the unmodified CPC, which lacked photothermal capability (Figure 9B).

**Figure 9:**
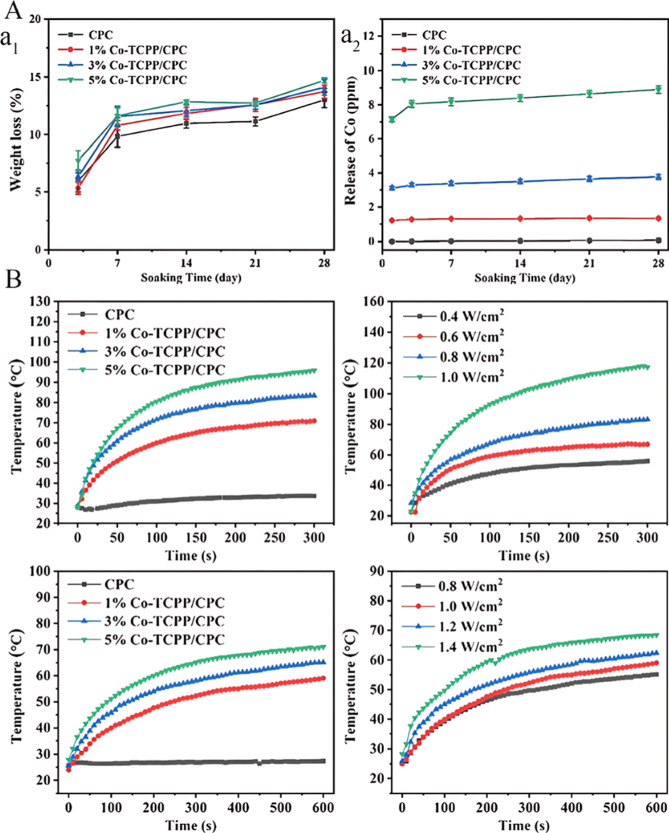
Fabrication of Co-TCPP/CPC to repair neoplastic bone defects. (A) In vitro degradation of bone cement. (a1) Weight loss of bone cements (a2) The release of Co2+ ions. (B) Photothermal performance197. Reproduced with permission from ref 196. Copyright 2021 Royal Society of Chemistry.

Cytotoxicity assays (CCK-8) on MG-63 bone tumor cells showed that Co-TCPP/CPC efficiently induced tumor cell death upon NIR irradiation while remaining nontoxic in the absence of light. In vivo studies using LM8 tumor-bearing nude mice confirmed that the composite cement generated significant localized hyperthermia under NIR light, promoting tumor apoptosis and necrosis (Figure 10A). Morphological analysis of rBMSCs cultured on the cement revealed that low cobalt ion concentrations enhanced cell proliferation and differentiation, whereas higher cobalt concentrations exerted cytotoxic effects [Figure 10(b_1_,b_2_)].

**Figure 10:**
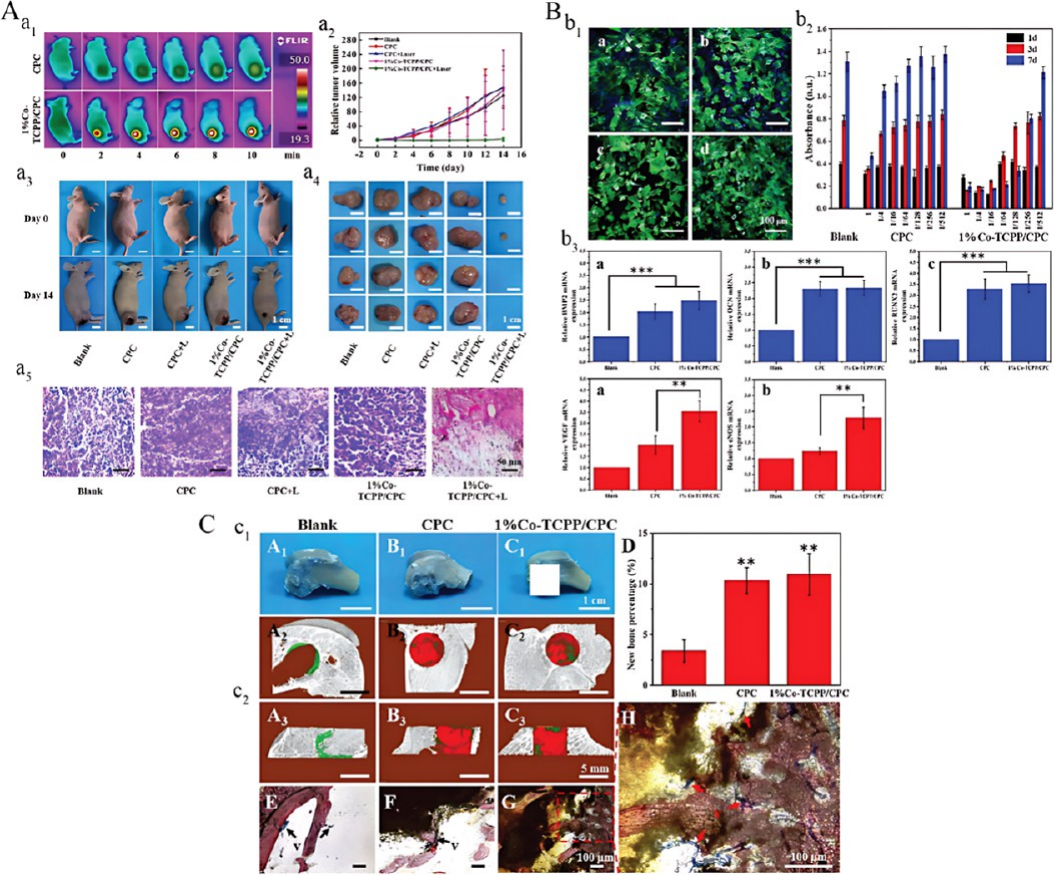
Biological analysis. (A) In vivo photothermal effect on tumor therapy after injection. (a_1_) Real-time temperature images of mice. (a_2_) Relative tumor volume. (a_3_) Photographs of nude mice. (a_4_) Tumor images. (a_5_) H&E staining. (B) In vitro osteogenic and angiogenic activity of bone cements. (b_1_) Confocal laser scanning microscopy images of rBMSCs. (b_2_) Proliferation of rBMSCs. (b_3_) Osteogenesis-related gene expression, including BMP2, OCN, and RUNX2 (the blue charts), and angiogenesis-related gene expression, including VEGF and eNOS (the red charts). (C) In vivo bone formation after bone cements filled in rabbit defects. (c_1_) Digital and (c_2_) micro-CT images of femoral defects. (c_3_) BV/TV. (c_4_) Histological analysis results of new bone.^196^ Reproduced with permission from ref 196. Copyright 2021 Royal Society of Chemistry.

The authors declared that expression levels of osteogenesis-related genes (BMP2, OPN, and RUNX2) in the 1%Co-TCPP/CPC group were comparable to those in the CPC group. while angiogenesis-related genes (VEGF and eNOS) were significantly upregulated in the Co-TCPP/CPC group. This upregulation of pro-angiogenic factors is consistent with cobalt’s ability to activate the HIF-1α pathway, thereby promoting endothelial cell proliferation and vascular stability. [Figure 10(b_3_)]. In vivo evaluation of a rabbit bone defect model confirmed that Co-TCPP/CPC is a promising bioactive material for promoting both bone regeneration and angiogenesis­(Figure 10C).^196^ Recent uses of cobalt-based MOFs for bone disease therapy are summarized in Table 6.

**Table 6:** Co-Based MOFs Studied in Bone Disease Treatment

MOF diversity	characteristics of MOFs	type of function	application	In vivo test	cell viability	cell name	method of synthesis	refs
TNT-ZIF-67@OGP	size of ZIF-67:168 ± 14 nm, size of osteogenic growth peptide-loaded MOFs: 189 ± 21 nm	implant	antibacterial, anti-inflammatory, Co2+ and osteogenic growth peptide delivery, biocompatible, osteogenesis	SD rats	Abs = 2.8 after 3 days	MSC, RAW264.7	electrochemical deposition method	^197^
Co-SIM-1	size: several microns	scaffold	Co^2+^ delivery, antibacterial	not mentioned	not mentioned	pseudomonas putida and S. aureus (bacteria)	electrospinning method	^198^
Co-TCPP/CPC	sheet size: about 400 nm, sheet thickness: 10–20 nm	Bone cement	photothermal, Co^2+^ delivery, antiosteosarcoma, osteogenesis, angiogenesis	female nude mice model for tumor therapy, New Zealand white rabbits for bone repair	toxic for MG-63, not toxic for RBMSCs	MG-63 for killing effect against tumor cells, RBMSCs for osteogenesis and angiogenesis	Co-TCPP nanosheet: ‘‘bottom-up’’ solvothermal method	^196^
GelMA@eIm/ZIF-67	particle size of eIm/ZIF-67 ≈200 nm-2 μm	bone filler	osteogenesis, ion delivery, angiogenesis, biocompatibility	calvarial defect model of SD rats	not mentioned	BMSCs, HUVECs	dispersion of eIm/ZIF-67 NPs in the GelMA solution	^199^

Based on the table evaluation of Co-MOFs in bone disease treatment, Co-TCPP/CPC emerges as the best candidate due to its multifunctional capacity demonstrated by photothermal antiosteosarcoma activity, Co^2+^ ion delivery, osteogenesis, and angiogenesis. This MOF effectively killed tumor cells in vivo while promoting bone repair with high cell viability in RBMSCs, indicating both safety and therapeutic efficacy. The TNT-ZIF-67@OGP implant also shows excellent prospects by combining antibacterial, anti-inflammatory, and osteogenic growth peptide delivery alongside cobalt ion release, proven to be biocompatible in rat models. It addresses both infection control and bone regeneration, critical for clinical bone defect scenarios. The GelMA@eIm/ZIF-67 bone filler holds solid promise due to its osteogenesis, ion delivery, and angiogenesis capabilities supported by in vivo calvarial defect models, reinforcing its role in bone healing and vascularization. Conversely, Co-SIM-1, while showing antibacterial and cobalt ion delivery capabilities, lacks in vivo and cellular viability data, making its translational potential limited at this stage.

Looking ahead, the multifunctionality of Co-MOFs positions them as highly adaptable platforms for complex bone disease treatment. Future work should focus on enhancing the controlled release of Co^2+^ and peptides, optimizing scaffold integration, and expanding rigorous in vivo and clinical validations to support safe, sustained therapy.

#### 4.1.8 The Role of Titanium and Ti-Based MOFs in Bone Regeneration

Titanium and its alloys have been extensively preferred for orthopedic implants owing to their superior biocompatibility, resistance to corrosion, and outstanding mechanical characteristics. Nonetheless, despite their broad application and clinical effectiveness, these implants frequently encounter issues such as failure, material degradation, and the necessity for revision surgeries, especially in patients presenting with low bone density, inadequate bone volume, or osteoporosis.^200,201^

The naturally occurring thin oxide film on titanium surfaces offers certain advantages but considerably restricts the bioactive potential that is essential for optimal tissue integration and regeneration. As a result, untreated titanium implants may lack the requisite bioactivity to achieve successful osseointegration. This drawback has prompted extensive investigation into various surface modification techniques aimed at enhancing the bioactivity of titanium implants.^201,202^ By modifying the surface physicochemical characteristics, the interactions at the implant interface can be optimized, thereby facilitating improved integration and minimizing the risks of peri-implant inflammation, bacterial infections, and compromised osteogenic capability.^203^

Surface modification and functionalization of titanium implants are strategic approaches designed to customize the surface structure and chemistry, thereby influencing initial cellular responses.^204^ Effective tissue-engineered bone regeneration necessitates the additional regulation of growth factors and osteogenic drugs. While trace amounts of these agents can promote osteogenic differentiation and bone formation, their uncontrolled release in vivo may result in adverse effects.^201,203^ Therefore, considerable attention has been directed toward developing titanium implants capable of the controlled delivery of such factors. To enhance biocompatibility, antibacterial properties, and osseointegration, various coating materials, including hydroxyapatite, bioactive molecules, and metal ions, have been applied.^205^

Titanium-based MOFs demonstrate superior biocompatibility and photocatalytic properties relative to other metals, positioning them as highly promising candidates for bone tissue engineering and nanodrug delivery applications. Titanium ions released from titanium-based implants, in conjunction with other osteoinductive ions such as strontium and zinc, synergistically stimulate angiogenesis by upregulating VEGF expression, thereby ensuring sufficient blood supply to regenerating tissues.^206^ A prominent example of such a material is MIL-125­(Ti), first described by Dan-Hardi et al. in 2009, which consists of titanium octahedra coordinated with terephthalate dianions, featuring accessible pore diameters of 6.13 and 12.55 Å. MIL-125­(Ti) has attracted considerable attention for its capacity to encapsulate small molecule drugs, including aspirin, ibuprofen, silver nanoparticles, and carbon monoxide gas, making Ti-MOFs an attractive platform for nanodelivery systems in medical contexts.^39^

#### 4.1.9 The Role of Lanthanum and La-Based MOFs in Bone Regeneration

Due to the growing ineffectiveness of traditional antibacterial agents caused by increasing drug resistance, research efforts have shifted toward rare earth elements, particularly lanthanum (La^3+^), for their promising antibacterial properties.^207,208^ Recently, lanthanum has attracted interest not only for its antimicrobial effects but also for its potential role in bone repair, leading to investigations into its use in composite coatings for orthopedic applications.^209^ La^3+^ promotes osteogenic differentiation and mineralization of BMMSCs by enhancing the expression of osteogenic markers like Runx2 and ALP. It also inhibits osteoclast differentiation and bone resorption by suppressing the nuclear factor-κB (NF-κB) signaling pathway, reducing bone loss.^210^ Additionally, La^3+^ enhances angiogenesis by promoting VEGF expression in HUVECs, supporting vascularized bone regeneration.^211^ This dual antibacterial and bone-regenerative functionality underscores the increasing importance of rare earth elements such as lanthanum in the development of advanced biomedical materials for orthopedic implants, paving the way for future research and technological progress in this field.

#### 4.1.10 The Role of Strontium and Sr-Based MOFs in Bone Regeneration

Strontium ranelate has been used therapeutically for the treatment of osteoporosis; however, its application is limited in patients with cardiovascular diseases due to associated side effects.^212^ Strontium influences both osteoblast and osteoclast activities, promoting bone formation and inhibiting bone resorption during the remodeling process. It has been incorporated as a doping element in bone substitutes, where controlled local release is crucial for treating osteoporotic bone conditions.^213^ The effect of strontium on the BMD remains somewhat controversial. Some studies report hypocalcemia and impaired bone mineralization with dietary strontium supplementation, whereas others demonstrate positive impacts on bone density, volume, and strength in animal models. Moreover, studies in monkeys and osteoporotic women have shown that strontium is highly incorporated into newly formed bone tissue, maintaining calcium content and mineralization without significantly increasing the risk of venous thromboembolism.^56^

Strontium enhances osteoblast activity and suppresses osteoclast activity primarily through mechanisms involving the Wnt/β-catenin signaling pathway. Animal studies have demonstrated that strontium upregulates extracellular matrix gene expression, thereby facilitating bone formation.^214^ Further research is needed to elucidate the molecular-level effects of strontium and its potential impacts on scaffolds and other tissue engineering applications. The beneficial effects of strontium on bone metabolism, despite its cardiovascular risks, suggest that local incorporation into bone substitutes, such as apatite coatings and bone cements, might offer a promising approach for osteoporotic patients. This method could leverage strontium ’s ability to locally regulate bone cell activities and support bone healing during physiological remodeling.^215^ The following demonstrates the other recent metal-based MOFs that were studied in bone disease treatment (Table 7).

**Table 7:** Other MOFs Studied in Bone Disease Treatment

MOF diversity	characteristics of MOFs	type of function	application	in vivo test	cell viability	cell name	method of synthesis	refs
MIL@Nd-HA/TiO2–Ti	surface area of MIL@Nd: 42.1 m2/g, average pore diameter of MIL@Nd: 8.8 nm	implant	biocompatibility, corrosion resistance, antibacterial, ion delivery, bioactivity	not mentioned	not mentioned	L929 mouse fibroblast cells	electrochemical method	^216^
MIL@La-HA/TiO2–Ti	surface area: 44.2866 m^2^/g; pore size: 8.2772 nm	implant	biocompatible, antibacterial, ion delivery	not mentioned	>100% after 48 h	L929 mouse fibroblast cells	electrochemical deposition method	^217^
MIL@Ce-HA/TiO2–Ti	surface area: 78.1089 m2/g; pore size: 6.6086 nm	implant	antibacterial, biocompatible, ion delivery	not mentioned	>100% after 72 h	L929 mouse fibroblast cells	electrochemical deposition method	^218^
Sr/HCOOH-MOF-ketoprofen	size: micrometer	carrier	drug delivery, anti-inflammatory, osteoarthritis treatment, biocompatible	not mentioned	not toxic	MG-63	Ketoprofen loading into MOF (conventional solution method and keeping in oil for a period of time)	^219^
Sr/PTA-MOF-Ketoprofen	size: micrometer	carrier	anti-inflammatory, osteoarthritis treatment, drug delivery	not mentioned	>90%	Chondrocyte	Ketoprofen loading into MOF (solvothermal method and Conventional solution method	^220^
Pt-MOF@Au@QDs/PDA	size: about 200 nm	nanoparticle	osteoarthritis treatment	DBA1/J mice, CIA mice mode	≈91%	RAW264.7, HFLS-RA cell	sonochemical synthesis method	^221^

## 5 Summary and Perspectives

MOFs have emerged as highly promising materials for bone defect repair and regeneration due to their unique structural and biological properties. The increasing prevalence of bone-related diseases and defects caused by aging, trauma, infection, tumors, and congenital abnormalities has driven extensive research into advanced biomaterials. Traditional materials such as ceramics, metals, polymers, composites, and natural biomaterials, while beneficial, present limitations including limited bioactivity, poor osteoinductivity, and the risk of infection.

MOFs, as inorganic–organic hybrid materials, offer distinct advantages such as adjustable pore size, high thermal stability, selective adsorption, and vast surface area. These features contribute to their multifunctional therapeutic capabilities, including bioactivity, corrosion resistance, biocompatibility, cellular proliferation, biodegradability, osteogenesis, angiogenesis, antibacterial activity, and controlled drug and ion delivery, which make them a greater substitute for repairing bone defects. These hybrid structures can serve as an implant, scaffold, hydrogel, nanoparticles, composite membrane, nanofiber, bone cement, and filler depending on the characteristics of metal ions, organic ligands, and other substances that are loaded or coated on their surfaces or pores.

This review comprehensively examines the potential of various MOFs incorporating diverse metal ions as effective promoters of bone regeneration and repair. Each MOF, characterized by its specific metal center and organic ligand, possesses unique properties that render it suitable for targeted therapeutic objectives. Notably, zinc ions have a well-established role in bone metabolism and are frequently incorporated into Zn-based MOFs to stimulate bone formation and inhibit osteoclast activity. Among these, ZIF-8 has garnered considerable research interest due to its distinct morphological features, multifunctionality, corrosion resistance, and advantageous biological properties, including bioactivity, biocompatibility, osteogenesis, and angiogenesis. Beyond ligand characteristics and surface modifications, the controlled, sustained release of metal ions from these frameworks is critical for therapeutic efficacy. For instance, the D-AHT system exemplifies a Zn-based MOF with robust drug and ion loading and release capabilities. Additionally, its favorable surface wettability enhances the adhesion and proliferation of relevant cell lines, such as MC3T3-E1 osteoblasts and HUVECs. The promotion of osteogenic and angiogenic activities in these systems is commonly evaluated through markers such as ALP activity and the expression of associated osteogenic and angiogenic genes, underscoring the multifunctional potential of metal-ion-based MOFs in bone tissue engineering applications.

The second most extensively studied metal ion after zinc, in the context of bone regeneration, is magnesium, primarily due to the pivotal role of Mg^2+^ ions as essential transporters in bone matrix synthesis with a density closely resembling that of natural bone. Mg-based MOFs significantly contribute to bone healing by promoting osteoblast proliferation and differentiation, thereby enhancing the regenerative process. An exemplar of such materials is the PLGA/Exo-Mg-GA MOF composite, which exhibits a strong interactive interface between the exosomes and the MOF matrix, as evidenced by zeta potential shifts and BET surface area analyses. The synergistic osteogenic, angiogenic, and anti-inflammatory effects observed in this system arise from the sustained release of Mg^2+^ ions and gallic acid, alongside the high surface area and unique nanostructure of the Mg-GA MOF. Despite these advantages, a notable limitation of Mg-based MOFs is their susceptibility to poor corrosion resistance in physiological fluids. To address this, researchers have developed hybrid metal coatings, such as Mg/Zn-MOF74, wherein the combination of Zn^2+^ and Mg^2+^ ions enhances aqueous stability. Furthermore, MOF74-modified samples demonstrate promising multifunctionality encompassing antibacterial, anti-inflammatory, and pro-osteogenic properties, underscoring their potential for advanced applications in bone tissue engineering.

Although Zr-based MOFs are generally considered less favorable compared to zinc- and magnesium-based MOFs, the C_2_S@PCN-224 composite represents a notable example where patients can benefit from the combined presence of Zr^2+^ and Ca^2+^ ions. This material integrates zirconium’s structural stability and calcium’s osteoconductive properties, fostering an environment conducive to bone regeneration. Experimental evaluations, including live/dead cell staining, ALP activity assays, and the upregulation of osteogenic-related gene expression, collectively indicate that modification with PCN-224 significantly enhances osteogenic differentiation. Such findings underscore the potential of this Zr- and Ca-based MOF system as a multifunctional scaffold that effectively promotes bone tissue repair by stimulating the key cellular responses necessary for regeneration.

Iron ions play a fundamental role in numerous cellular processes critical to human physiology, including the synthesis of DNA and RNA, protein production, electron transport, cellular proliferation, and differentiation. Within the context of bone regeneration, iron ions incorporated into Fe-MOFs facilitate angiogenesis, a vital process responsible for delivering nutrients and oxygen to healing bone tissue. One notable example is the MOF@HA@PCA system, a pH-responsive, controlled drug release carrier designed for the treatment of OA. This carrier has demonstrated significant therapeutic efficacy in both in vitro and in vivo studies, highlighting its potential for modulating inflammation and promoting tissue repair through targeted delivery and sustained release of active agents.

Cu-MOFs have gained considerable attention for their role in repairing bone defects, particularly due to their intrinsic photothermal properties. Cu-MOFs exhibit potent antibacterial effects, which are crucial for reducing infection risks during the bone healing process. A prominent concern following surgical tumor resection is the recurrence of bone tumors; hence, materials capable of both eradicating residual tumor cells and facilitating bone regeneration have become a key focus in biomaterial research. To address this, Cu-TCPP-TCP nanosheets were developed via integration with 3D printing technology. This highly engineered scaffold demonstrates effective photothermal ablation of LM8 tumor cells by elevating local temperatures under NIR light irradiation, achieving tumor cell destruction without inducing toxicity in the surrounding healthy tissues. The dual function of tumor eradication and bone regeneration rendered by these Cu-based nanosheets represents a promising approach for postsurgical management of bone cancer and subsequent defect repair.

Though cobalt ions are considered less prominent among metal ions used in MOFs for bone disease treatment, their biological significance remains crucial, particularly in stimulating red blood cell production and promoting angiogenesis through the activation of HIF. A representative example is Co-TCPP/CPC cement, developed for repairing neoplastic bone defects. This multifunctional biomaterial exhibits selective cytotoxicity against bone tumor cells upon NIR irradiation via photothermal effects, while remaining nontoxic in the absence of such stimulation. In addition to cobalt, other metal ions, including strontium, titanium, lanthanum, platinum, cerium, and manganese, have been incorporated into MOFs, broadening the therapeutic functionalities and enabling tailored approaches for bone disease management. The identification of a single, definitive MOF that can be considered the optimal material for bone regeneration remains a complex and challenging endeavor due to the multifaceted nature of bone healing processes. However, Zn-based MOFs have emerged as the most extensively studied and promising candidates in the context of bone disease treatment. Both in vitro and in vivo investigations have consistently demonstrated the high efficacy of Zn-based MOFs in stimulating osteogenesis, enhancing mechanical strength, and promoting bone repair. While certain metal ions exhibit particularly notable potential, it is critical to recognize that the intricate process of bone regeneration necessitates a comprehensive consideration of the diverse roles played by various metal ions. Additionally, critical physicochemical characteristics of MOFs, including particle size, crystal morphology, surface charge, wettability, and pore size, exert a profound influence on their therapeutic performance. Consequently, future MOF designs for bone repair are anticipated to evolve toward multi-ion frameworks capable of codelivering bioactive molecules or drugs with direct osteoinductive functions, thereby maximizing regenerative outcomes. With regard to future developments, a mixed magnesium–copper MOF will demonstrate enhanced adhesion, proliferation, and differentiation of osteogenic cells, benefiting from the copper component’s significant antimicrobial activity, which plays a crucial role in preventing implant infections and improving postoperative recovery. Additionally, cerium/strontium bimetallic MOF coatings applied to titanium implants suggest antioxidant activities that effectively reduce oxidative stress. This reduction in oxidative stress aids in restoring cellular function and significantly promotes new bone formation and osteointegration. Besides, hybrid Ca/Mg-MOFs are recommended specifically for bone regeneration applications since osteogenic and biomineralization capabilities of calcium, along with harnessing the angiogenic and proliferative effects of magnesium, thereby offering a synergistic approach to enhance bone repair and healing.

Overall, addressing these multidisciplinary challenges through collaborative research in materials science, biology, and clinical medicine will be crucial to harnessing the multifunctional potential of MOFs. Such efforts will enable the development of safe, effective, and personalized therapies that overcome the limitations of current bone regeneration strategies and improve patient outcomes.

## Abbreviations

ALP: Alkaline phosphatase
AXIN2: Axis inhibition protein 2
AHT: Heat-treated titanium
AT: Alkali-heat-treated titanium
ACAN: Aggrecan
Adamts5: A disintegrin and metalloproteinase with thrombospondin motifs 5
AFM: Atomic force microscopy
Aln: Alendronate
Alen: Alendronate
BTE: Bone tissue engineering
BMD: Bone mineral density
BMMSCs: bone marrow mesenchymal stem cells
BMC: Bone mineral content
BMPs: Bone morphogenetic proteins
BALB/c mice: Bagg Albino, Laboratory-bred strain of the house mouse
BV/TV: Trabecular bone volume to total volume fraction
BG: Bioglass
BET: Brunauer–Emmett–Teller
CAM: Chick embryo chorioallantoic membrane
Col2A1: type II collagenopathies in a Chinese male
COX2: cyclooxygenase 2
chondrocytes: the cartilage that is solely composed of cells
CPC: calcium phosphate cements
CCK-8: Cell Counting Kit-8
Co-SIM-1: Cobalt-based substituted imidazolate
CIA mice: Collagen-induced arthritis
CA-CS: Catechol-chitosan
Col1: Collagen type I
Col: Collagen
CaP: calcium phosphate
CaSR: Calcium-sensing receptor
CHI: Chitosan
C_2_S: β-Ca_2_SiO_4_
CCR7: CC Chemokine receptor 7
CD31: cluster of differentiation 31
DMOG: dimethyloxalylglycine
D-AHT: dimethyloxalylglycine into zeolitic imidazolate framework-8-modified implants
DCPD: dicalcium phosphate dihydrate
DEX: dexamethasone
DOX: doxorubicin
DAPI: 4′, 6′ -diaminido-2-phenylindole
d-Arg: d-arginine
DLS: dynamic light scattering
DFT: density functional theory
*E. coli*: *Escherichia coli*
Enos: endothelial nitric oxide synthase
eIm: 2-ethylimidazole
ECM: Extracellular matrix
EC50: half maximal effective concentration
ERK: extracellular signal-regulated protein kinases
EDS: energy-dispersive X-ray spectroscopy
FA: folic acid
FITC: fluorescein isothiocyanate
FOS: Fosfomycin
FT-IR: Fourier-transform infrared
FE-SEM: field emission scanning electron microscopy
GAG: glycosaminoglycan
GelMA: gelatin methacrylate
GA: gallic acid
GLUT: glucose transporter
GPX1: glutathione peroxidase 1
Hmsc: Human mesenchymal stem cell
hBMSCs: Human bone marrow-derived mesenchymal stem cells
HUVECs: Human umbilical vein endothelial cells
HADMSCs: Human adipose tissue-derived mesenchymal stem cells
HDPSCs: human dental pulp stem cells
HA: hyaluronic acid
HE: hematoxylin and eosin
H & E stain: hematoxylin and eosin stain
HBMSCs: human bone marrow stromal cells
HIF: hypoxia-inducible transcription factors
Hap: hydroxyapatite nanoparticles
HFLS-RA: human fibroblast-like synoviocytes: rheumatoid arthritis
HKUST: Hong Kong University of Science and Technology
Hfob: Human fetal osteoblastic
IA: Intra-articular
IL6: Interleukin 6
iNos: Inducible nitric oxide synthase
ICP-AES: inductively coupled plasma–atomic emission spectrometry
IRMOF: IsoReticular metal–organic frameworks
ICA: Icariin
IP_3_: Inositol 1,4,5-trisphosphate
KDR: Kill-to-death ratio
Ket: Ketoprofen
LRP5: Low-density lipoprotein receptor-related protein 5
LM8: Lilama 18 Joint Stock Company
LBL: layer-by-layer
Levo: Levofloxacin
LIG: Lignin
MG-63: fibroblasts osteosarcoma cells
MBMSCs: mouse bone marrow stem cells
M-CSF: Macrophage colony-stimulating factor
MAPK: Mitogen-activated protein kinase
MTT assay: 3-[4,5-dimethylthiazol-2-yl]-2,5 diphenyl tetrazolium bromide) assay
Mmp1: matrix metallopeptidase 1
Micro-CT: microcomputed tomography
MOF: metal organic framework
MIL: Materials of Institute Lavoisier
MPCs: magnesium phosphate bone cement
M-CSF: macrophage colony-stimulating factor
MBG: bioactive glass
mRNA: messenger ribonucleic acid
NF-κB: nuclear factor-κB
NIR: near infrared
NFATc1: nuclear factor of activated T cell 1
NRF2: nuclear factor erythroid 2–related factor 2
OA: osteoarthritis
OARSI: Osteoarthritis Research Society International
OCN: Oncology Certified Nurse
OGP: Osteogenic growth peptide
OPN: Osteopontin
Opg: Osteoprotegerin
OA: Osteoarthritis
PCA: Protocatechuic acid
PLA: polylactic acid
PRGF: Plasma rich in growth factors
PDA: Polydiacetylene
PLLA: poly-l-lactic acid
PDA: polydopamine
PCL: polycaprolactone
PG: polycaprolactone/gelatin
PDGF: platelet-derived growth factor
PLGA-TCP: poly­(lactide-co-glycolide)-tricalcium phosphate
PP: polypropylene
PLGA: poly­(lactic acid-co-glycolic acid)
PBS: phosphate buffer saline
PT: porous titanium
PTH: parathyroid hormone
PLC: phospholipase C
PAA: Poly­(acrylic acid)
PEO: poly ethylene oxide
PIII: plasma immersion ion implantation
QDs: quantum dots
Raw264.7: Mouse macrohage cells
RBMSCs: rat bone marrow mesenchymal stem cells
Rat RCT model: rat rotator cuff tear model
Rob cells: rat osteoblast cells
RMSCs: rat mesenchymal stromal cells
RANKL: receptor activator of nuclear factor kappa-Β ligand
RUNX2: runt-related transcription factor 2
Ral: raloxifene
ROS: reactive oxygen species
SMCs: smooth muscle cells
SD: Sprague–Dawley
*S. aureus*: *Staphylococcus aureus*
SaOS-2: cell line derived from primary osteosarcoma
SEM: scanning electron microscope
SMCs: smooth muscle cells
SF: silk fibroin
Saos: sarcoma osteogenic
SOCE: store-operated calcium entry
SOD-1: Cu/Zn superoxide dismutase
TCPs: tricalcium phosphates
TRAP: tartrate-resistant acid phosphatase
TCPP: tetrakis (4-carboxyphenyl) porphyrin
TEM: transmission electron microscopy
TNTs: titanium dioxide nanotubes
TGF-β: transforming growth factor-β
TGF-α: transforming growth factor alpha
Tenogenesis: tendon and bone regeneration
TGA: thermogravimetric analysis
UV: ultraviolet
UiO: University of Oslo
VE-cad: vascular endothelial cadherin
VEGF: vascular endothelial growth factor
VAN: vancomycin
WCAs: water contact angle
Wnt: wingless-related integration site
XRD: X-ray diffraction
ZIF: zeolitic imidazolate framework
Z-AHT: zeolitic imidazolate framework-8-modified implants
β-CD: β-cyclodextrin.
